# CASK promotes non-small cell lung cancer growth through coordinated regulation of EGFR expression, trafficking, and p21 expression

**DOI:** 10.1186/s12929-026-01252-z

**Published:** 2026-05-09

**Authors:** Yun-Han Lai, Duen-Yi Huang, Yen-Yu Chang, Yen-Lin Huang, Wan-Wan Lin

**Affiliations:** 1 Department of Pharmacology, College of Medicine, National Taiwan University, Taipei, 100233 Taiwan; 2 Department of Pathology, National Taiwan University Cancer Center, Taipei, 100233 Taiwan; 3 Graduate Institute of Medical Sciences, Taipei Medical University, Taipei, 110301 Taiwan

**Keywords:** CASK, NSCLC, p21, p53, EGFR, ERK, Tumor growth

## Abstract

**Background:**

Calcium/calmodulin-dependent serine protein kinase (CASK), a member of the membrane-associated guanylate kinase (MAGUK) family, functions as a multifunctional scaffold protein by engaging diverse binding partners. Although CASK has been implicated in tumorigenesis, its molecular role in the pathogenesis of non-small cell lung cancer (NSCLC) remains poorly characterized.

**Methods:**

CASK expression was assessed by immunohistochemistry in lung adenocarcinoma tissues and matched non-tumor samples. Bioinformatics analyses were performed using Gene Expression Omnibus (GEO) and The Cancer Genome Atlas (TCGA) datasets. Functional studies were conducted in epidermal growth factor receptor (EGFR) wild-type H1299 cells and EGFR exon 19-deleted PC9 cells following CASK silencing. RNA-Seq was used to identify differentially expressed genes, and assays of cell growth, cell cycle progression, EGFR trafficking, and downstream signaling were performed.

**Results:**

CASK expression was significantly elevated in early-stage NSCLC, and higher levels correlated with reduced overall and first progression survival. Silencing CASK inhibited cell growth by blocking the G1-S transition and inducing p21waf1/Cip1, a key mediator of cell cycle arrest, at both transcript and protein levels. Growth suppression was rescued by treatment with a p21 inhibitor UC2288, confirming its central role. Elevated p21 in CASK-deficient cells was reversed by EGFR, AKT, and ERK inhibitors, suggesting that CASK is involved in downregulating p21 via suppressing EGFR/ERK-dependent transcription and EGFR/AKT-dependent protein stabilization. Although p53 is constitutively involved in upregulation of p21 gene expression, the findings from the p53 inhibitor and p21 promoter activity assay excluded the role of p53 in CASK silencing-induced p21 upregulation. Furthermore, CASK modulated the autocrine EGFR loop. CASK silencing enhanced EGFR expression through gene transcription and post-transcriptional protein stabilization and promoted EGFR activation by upregulating TGF-α in p53-, ERK-, and AKT-dependent manners. Notably, CASK loss did not affect EGFR trafficking to early endosomes but delayed its transition to late endosomes in an ERK-dependent fashion, thereby reducing receptor degradation.

**Conclusions:**

We identified CASK as a previously unrecognized driver of NSCLC growth and a potential prognostic biomarker. By regulating EGFR trafficking to late endosomes and attenuating AKT and ERK signaling, CASK suppresses p21 expression and promotes NSCLC cell proliferation, revealing a novel proliferation regulator in NSCLC.

**Supplementary Information:**

The online version contains supplementary material available at 10.1186/s12929-026-01252-z.

## Background

Lung cancer is the most commonly diagnosed cancer and the leading cause of cancer-related mortality worldwide. Owing to its high incidence and mortality, the overall 5-year survival rate of lung cancer remains substantially lower than that of many other major cancers [1]. Calcium/calmodulin-dependent serine protein kinase (CASK), the mammalian homolog of *Caenorhabditis elegans* LIN-2, is a multidomain scaffolding protein that belongs to the membrane-associated guanylate kinase (MAGUK) family. Structurally, CASK contains, from the N- to the C-terminus, a CaMK (Ca^2^⁺/calmodulin-dependent protein kinase), followed by L27 (comprising L27A and L27B subdomains), PDZ, SH3 (Src homolog 3), and GUK (guanylate kinase) domains. The mutations of the *CASK* gene have been linked to X-linked intellectual disability [2]. In neurons, CASK performs diverse regulatory functions through interactions with multiple binding partners. For example, CASK interacts with liprin via its CaMK and L27A domains to regulate synaptic vesicle cycling at presynaptic sites [3], and it co-localizes with Parkin at postsynaptic sites through its PDZ domain, contributing to synaptic transmission and plasticity [4]. In addition, CASK can translocate to the nucleus, where it associates with the transcription factor Tbr-1 to regulate neuronal development [5].

CASK is expressed in various cancer tissues and has been implicated in tumor malignancy. Elevated CASK expression is associated with advanced tumor progression, enhanced migration, poor prognosis, and reduced overall survival (OS) in gastric, colorectal, pancreatic, and prostate cancers [6–9]. In hepatocellular carcinoma (HCC), CASK has also been reported to promote sorafenib resistance by enhancing JNK–mediated autophagy [10]. In contrast to its proliferative role in cancer cells [8–10], CASK negatively regulates cell-cycle progression in epidermal keratinocytes [11]. Knockdown of CASK in keratinocytes accelerates cell-cycle progression. It is accompanied by Myc upregulation and hyperphosphorylation of retinoblastoma protein Rb, as well as an exaggerated hyperproliferative response to keratinocyte growth factor and transforming growth factor-α (TGF-α) [11]. p21waf1/Cip1 is a cyclin-dependent kinase (CDK) inhibitor that suppresses cyclin–CDK complex activity and serves as a principal downstream effector of p53. In endothelial cells, CASK has been shown to enhance p21 gene promoter activity by binding to Id1, thereby alleviating Id1-mediated repression of E2A and facilitating E2A-dependent p21 gene transcription [12]. Collectively, these findings suggest that CASK modulates cell growth in a highly cell-type-specific manner.

Epidermal growth factor receptor (EGFR), a member of the ErbB family, is a transmembrane receptor tyrosine kinase. Upon binding to its cognate ligands, EGFR undergoes dimerization, autophosphorylation at multiple tyrosine residues, and subsequent endocytosis [13]. In *C. elegans*, CASK/LIN-2 recruits EGFR substrate protein 8 (EPS-8) to the basolateral plasma membrane, thereby stabilizing EGFR and preventing its rapid internalization and degradation [14, 15]. EGFR activation has been shown to induce p21 gene expression in multiple cell types, including fibroblasts [16], A431 cells [17], squamous carcinoma cells [18], enterocytes [19], head and neck cancer cells [20], HCC [21], and glioblastoma cells [22].

To date, the role of CASK in NSCLC progression has not been investigated. Given that NSCLC progression and resistance to targeted therapies are highly dependent on constitutive EGFR activity [23, 24], we sought to elucidate the role of CASK in NSCLC, with particular emphasis on the signaling pathways linking EGFR to p21. Our findings identify CASK as a tumor-promoting factor in NSCLC. CASK enhances lung cancer cell growth by suppressing p21 gene transcription and p21 protein stability through inhibiting the EGFR–ERK and EGFR-AKT signaling pathways, respectively. Moreover, CASK downregulates EGFR signaling, at least in part, through reduced ERK-, AKT-, and p53-dependent EGFR ligand expression and increased EGFR trafficking-associated degradation via inhibition of ERK.

## Material and methods

### Reagents and antibodies

RPMI-1640, Opti-MEM, fetal bovine serum (FBS), and trypsin-EDTA were bought from Gibco (Carlsbad, CA, USA). Penicillin-streptomycin and penicillin-streptomycin-amphotericin B solutions were purchased from Biological Industries (Kibbutz Beit Haemek, Israel). Sodium bicarbonate was obtained from Honeywell (Morris Plains, NJ, USA). Dulbecco’s phosphate-buffered saline (PBS), NR162, MG132, and pifithrin-α (PFT-α) were bought from Sigma-Aldrich (St. Louis, MO, USA). Recombinant human EGF was purchased from PeproTech (Rocky Hill, NJ, USA). Cycloheximide, U0126, and bafilomycin A1 (BafA1) were ordered from Calbiochem (San Diego, CA, USA). PI/RNase staining buffer was obtained from BD Biosciences (#550825, San Jose, CA, USA). UC2288 (a p21 inhibitor, HY-112780), AKT inhibitor VIII (HY-10355, AKTI), and epothilone B *(*EpoB) were obtained from MedChemExpress (HY-17029, Monmouth Junction, NJ, USA). Vincristine was purchased from Cayman (#2068-78-2, Ann Arbor, MI, USA). Gefitinib (ZD1839) was purchased from Selleckchem (#184475-35-2, Houston, TX, USA). Lipofectamine^™^ 2000 transfection reagent was ordered from Invitrogen (#11–668-019, Carlsbad, CA, USA). Antibodies against p-EGFR (Y1068, #2234), p-ERK (#9101), p-Akt (S473, #9271), Akt (#9272), EEA1 (#3288S), and Rab7 (#9367S) were purchased from Cell Signaling (Beverly, MA, USA). Antibodies of CASK (sc-13158), ERK (sc-271269), EGFR (sc-120), p21 (sc-53870), β-actin (sc-47778), cyclin B (sc-166210), CDK1 (sc-53219), cyclin D1 (sc-718), and acetylated α-tubulin (sc-23950) were obtained from Santa Cruz (Santa Cruz, CA, USA). Antibodies of p-histone H3 (Ser10, GTX128116), histone H3 (GTX122148), and MDM2 (GTX100653) were purchased from GenTeX (Irvine, CA, USA). Antibody of p53 (PAb1801) was purchased from Merck Millipore (#D54720, Burlington, MA, USA).

### Cell lines and cell culture

Human NSCLC adenocarcinoma cell lines H1299 (EGFR wild type) and PC9 (EGFR exon 19 deletion) were cultured in RPMI-1640 medium supplemented with 10% FBS and 1% penicillin-streptomycin-amphotericin B solution. PC9 cells were kindly provided by Dr. Chih-Hsin Yang (Graduate Institute of Oncology, Cancer Research Center, National Taiwan University), and H1299 cells were obtained from American Type Culture Collection (Manassas, VA, USA).

### Construction of CASK silencing stable cell lines using lentiviral transfection

H1299 and PC9 cells were seeded in 6-well plates at a density of 2 × 10^5^ cells per well. After overnight incubation, cells were infected with lentiviral particles expressing shRNA targeting CASK (shCASK; TRCN195100, 8.3 × 10^4^ RIU/μl) at a multiplicity of infection (MOI) of 7.5, or with control shRNA lentivirus (shCTL; TRCN72249/C6-4-1.1.1) obtained from the RNAi Core Facility, Academia Sinica, in the presence of polybrene (8 μg/ml). After 24 h of infection, the medium was replaced with fresh complete medium containing puromycin (3 μg/ml) for 1 week to select stably transduced cells, followed by maintenance in medium containing puromycin (1 μg/ml). Knockdown efficiency was confirmed by Western blot analysis of CASK expression.

### Cell apoptosis assay

Cell viability was assessed using an Annexin V/propidium iodide (PI) apoptosis detection kit (#640914, BioLegend, San Diego, CA, USA) according to the manufacturer’s instructions. Cells were seeded in 6-well plates at a density of 1 × 10^5^ cells per well and incubated overnight at 37 °C in a humidified atmosphere containing 5% CO_2_. Following treatment with the indicated concentrations of reagents for specified time periods, cells were harvested, and the cell pellet was resuspended in 100 μl of binding buffer. Cells were then stained with 5 μl of Annexin V and 10 μl of PI for 15 min at room temperature in the dark. Cell viability and apoptosis were analyzed by flow cytometry using a FACSCalibur system (BD Biosciences, San Jose, CA, USA), and data were processed with FlowJo software.

### Immunohistochemical staining of CASK in tumor and non-tumor tissues from lung adenocarcinoma patients

CASK protein expression in lung adenocarcinoma specimens was analyzed using protocols approved by the Institutional Review Board of National Taiwan University Hospital (approval no. 202206034RIND). Paraffin-embedded tissue specimens were retrospectively collected for immunohistochemical analysis, and all procedures were conducted in accordance with the principles of the Declaration of Helsinki. Formalin-fixed, paraffin-embedded tissue sections were deparaffinized and rehydrated, followed by heat-induced antigen retrieval in EDTA buffer (pH 6.0) at 95 °C for 92 min. Sections were then incubated with an anti-CASK primary antibody at a dilution of 1:50 (#sc-13158, Santa Cruz, CA, USA) at 37 °C for 2 h. Antigen detection was performed using the OptiView DAB IHC Detection Kit (Roche, Mannheim, Germany) according to the manufacturer’s instructions, and sections were counterstained with hematoxylin. CASK expression levels in lung adenocarcinoma cells were evaluated based on immunostaining intensity.

### RNA-sequence (RNA-Seq) analysis

Total RNA was extracted using TRIzol reagent (PT-KP200CT, PROtech, Rosemont, IL, USA) according to the manufacturer’s instructions. Functional gene annotation and enrichment analyses were performed using Gene Ontology (GO) and Kyoto Encyclopedia of Genes and Genomes (KEGG) pathway analyses with the ClusterProfiler package (https://github.com/YuLab-SMU/clusterProfiler).

### Analysis of the CASK gene expression in the cancer genomics dataset

CASK gene expression data were obtained from the GEO and TCGA datasets [25]. The association of CASK expression with patient OS and first progression (FP) was analyzed using the Kaplan-Meier Plotter [26]. CASK expression across different stages of lung cancer was evaluated using the Gene Expression Profiling Interactive Analysis (GEPIA) platform [27].

### Analysis of CASK mutant gene distribution

The distribution of CASK gene mutations in lung cancer, as well as patient survival data comparing normal and mutant CASK groups, were obtained from the Catalogue of Somatic Mutations in Cancer (COSMIC) and cBioPortal [28].

### Colony formation assay for cell proliferation

A total of 500 cells per well were seeded in 6-well plates. Cells were cultured for 10 days, with the medium replaced every three days. Colonies were then fixed with methanol for 10 min at room temperature and stained with 0.5% (w/v) crystal violet in distilled water for 30 min at room temperature. Excess stain was removed by washing three times with water, and colonies were counted. To quantify colony staining, 1 ml of 10% acetic acid was added to each well, and absorbance was measured at 595 nm. Wells containing 10% acetic acid only were used as blanks.

### Bromodeoxyuridine (BrdU) assay

After overnight serum starvation, cells were incubated for the indicated times, and cell proliferation was assessed using a BrdU ELISA kit (#11669915001, Roche, IN, USA) according to the manufacturer’s instructions. Briefly, 10 μl of BrdU labeling solution was added to each well for 4 h, and BrdU incorporation was detected using a BioTek Synergy H1 microplate reader.

### Cell cycle assay

Cells (2 × 10^5^ per well) were seeded in 6-well plates with serum-free RPMI-1640 medium. After overnight adherence, the medium was replaced with fresh complete medium for the indicated times. Cells were then trypsinized, collected by centrifugation at 1,100 rpm for 5 min, and fixed in 75% precooled ethanol overnight at − 20 °C. After centrifugation, the supernatant was discarded, and the cell pellet was washed twice with PBS. Cells were resuspended in 500 μl of PI/RNase staining buffer (#550825, BD Biosciences, San Jose, CA, USA) and incubated for 30 min at room temperature in the dark. Cell cycle distribution was analyzed by flow cytometry using a FACSCalibur system from BD Biosciences (San Jose, CA, USA), and data were processed with ModFit LT™ software.

### Plasmid transfection for CASK overexpression

Cells were seeded in 6 cm dishes at a density of 1 × 10^6^ cells per dish and allowed to adhere overnight. CASK plasmid DNA (8 μg) and Lipofectamine 2000 (20 μl) were each separately diluted in 500 μl of Opti-MEM medium. The diluted DNA and Lipofectamine 2000 were then combined and incubated at room temperature for 20 min to allow complex formation. The mixture was added to the cells, which were incubated for 24 h at 37 °C. Transfection efficiency was confirmed by Western blot analysis.

### Immunoblotting analysis

Cells were seeded in a 12-well plate at a density of 2 × 10^5^ cells/well overnight. After different treatments for indicated time, the medium was aspirated and cells were lysed by 50 μl of RIPA buffer (50 mM Tris,150 mM NaCl, 0.1% SDS, 0.1% NaDOC, 1 mM PMSF, 2 mM EDTA, 2 mM NaF, 2 mM Na_3_VO_4_, 1% Triton X-100, 1:250 protease inhibitor cocktail) on ice for 30 min. The lysates were centrifuged at 12,000 rpm at 4 ℃ for 25 min, and the supernatant was collected. The concentration of lysate protein was measured by bovine serum albumin (BSA) standard assay with Protein assay dye reagent (#5000006, Bio-Rad, Hercules, CA, USA). An equal amount of protein sample (30 μg) was separated by 8**–**15% SDS-PAGE gel electrophoresis and transferred to a 0.45 μm PVDF. After blocked by 5% skim milk at room temperature for 1 h, the membranes were incubated overnight at 4 ℃ with specialized primary antibodies at a dilution of 1:1000. Then the membranes were washed with 1 × TBST, containing 0.1% (v/v) Tween-20 (P2287, Sigma-Aldrich, St. Louis, MO, USA) three times and incubated with HRP-conjugated anti-mouse or anti-rabbit secondary antibodies at a dilution of 1:5000 for 1 h at room temperature. After washing three times with 1 × TBST, the proteins were visualized by using an enhanced chemiluminescence kit (WBKLS0500, Millipore, Burlington, MA, USA) and analyzed by Image Lab software 6.0.1 system (Bio‑Rad Laboratories, Inc., Hercules, CA, USA). Immunoreactive bands were quantified by using ImageJ software.

### p21 promoter luciferase assay

Cells were co-transfected with the p21 reporter plasmid and a β-galactosidase expression vector (pCMV-β-Gal) using Lipofectamine 2000 (Invitrogen, Carlsbad, CA, USA). After treatment with the indicated reagents, cells were lysed in reporter lysis buffer, and the lysates were incubated with luciferase substrate from the luciferase assay system (Promega, Madison, WI, USA). Luciferase activity was measured using a microplate luminometer (Packard, Meriden, CT, USA). Relative luciferase activity was normalized to β-galactosidase expression to account for transfection efficiency and is presented as a percentage of the control group. The p21 reporter constructs—including the full-length (− 2300 to + 8), p53-binding-site-deleted (− 1866 to + 8), and minimal (− 180 to + 8) promoters-were kindly provided by B. Vogelstein (Johns Hopkins University, Baltimore, MD, USA).

### Real-time polymerase chain reaction (RT-PCR)

Total RNA was extracted by homogenizing cells in 300 μl of TRIzol reagent (#PT-KP200CT, PROtech, Rosemont, IL, USA) on ice, followed by phase separation with chloroform and precipitation with ice-cold isopropanol at − 20 °C overnight. For reverse transcription (RT), 1–2 μg of RNA was mixed with 0.5 μl of random primers (500 μg/ml; #C118A, Promega, Madison, WI, USA) and incubated at 70 °C for 5 min. The reaction mixture was then supplemented with 5 μl of 5X RT buffer, 2.5 mM dNTPs, 1 μl of MMLV reverse transcriptase (Promega, M170A), and 0.5 μl of RNase inhibitor (Promega, N211A), followed by incubation at 37 °C for 1–2 h and enzymatic inactivation at 90 °C for 5 min. Quantitative PCR (qPCR) was performed in a 25 μl reaction containing 2.5 μl of cDNA template, 0.25 μl each of forward and reverse primers (30 μM), 9.5 μl of DEPC-treated water, and 12.5 μl of 2X SYBR Green Master Mix (#4367659, Applied Biosystems, Thermo Fisher Scientific, Waltham, MA, USA). Primer sequences are listed in Supplementary Table 1. Gene expression levels were normalized to β-actin as an internal control. The cycling conditions were as follows: initial incubation at 50 °C for 2 min and 95 °C for 10 min; 40 cycles of 95 °C for 15 s and 60 °C for 1 min; followed by melt curve analysis at 90 °C for 15 s and 60 °C for 1 min.

### Flow cytometry for surface EGFR expression

Cells (1 × 10^5^/well) were seeded in 6-well plates and cultured in medium with or without serum. Serum-starving cells were stimulated with EGF (50 ng/ml) for the indicated time points. Cells were harvested by trypsinization and centrifuged at 1,100 rpm for 5 min in 1.5 ml microtubes. The supernatant was aspirated, and cells were resuspended in 0.5% BSA in PBS and incubated on ice for 30 min for blocking. Following centrifugation at 1,100 rpm for 5 min, the blocking solution was removed, and cells were incubated with 0.8 μg/ml Alexa Fluor^®^ 488-conjugated anti-human EGFR antibody (#352908, BioLegend, San Diego, CA, USA) diluted in 0.5% BSA. An Alexa Fluor^®^ 488-conjugated anti-mouse IgG F(ab’)₂ fragment (#4408, Cell Signaling Technology, Beverly, MA, USA) was used as a negative control. After incubation on ice for 30 min, cells were washed by centrifugation at 1100 rpm for 5 min and resuspended in 500 μl PBS prior to transfer to flow cytometry tubes. Surface EGFR fluorescence was analyzed using a FACSCalibur flow cytometer (BD Biosciences, San Jose, CA, USA), and data were processed with FlowJo software.

### Immunofluorescence

Cells were seeded onto coverslips in 24-well plates at a density of 2 × 10^5^ cells per well and cultured overnight. Cells were serum-starved for 24 h and then stimulated with EGF (50 ng/ml) for the indicated time points. Cells were fixed with 500 μl of 4% paraformaldehyde in PBS for 20 min at room temperature, followed by three washes with 500 μl PBS. Cells were permeabilized with 500 μl of 0.1% Triton X-100 in PBS for 15 min at room temperature, then washed 3 times with PBS. Blocking was performed with 500 μl of 5% BSA in PBS for 2 h at room temperature. Cells were then incubated with primary antibody (1:200 dilution in PBS containing 0.1% BSA) at 4 °C overnight. After three washes with PBS, cells were incubated with fluorescence-conjugated secondary antibody (1:2000 dilution in PBS containing 0.1% BSA) for 45 min at room temperature in the dark. Cells were washed three times with PBS, and coverslips were mounted onto glass slides using mounting medium containing DAPI for nuclear staining. Fluorescence images were acquired using a Zeiss ApoTome.2 microscope and analyzed with ZEN Blue 2.6 software.

### Co-immunoprecipitation

Cells were seeded in 6 cm dishes at a density of 1 × 10^6^ cells per dish and incubated at 37 °C in 5% CO_2_ overnight. Cells were lysed in 500 μl of RIPA buffer and sonicated for 10 s until fully homogenized. Lysates were centrifuged at 12,000 rpm at 4 °C for 25 min, and the supernatant was collected. Equal amounts of protein (500 μg) were adjusted to a final volume of 500 μl with RIPA buffer. Samples were precleared by incubation with 10 μl of protein A beads and 1 μl of normal IgG antibody (sc-2025, 400 μg/ml) for 30 min at 4 °C. After centrifugation at 7,000 rpm for 5 min, the pellet was discarded, and the supernatant was transferred to a fresh microcentrifuge tube. For immunoprecipitation, 1 μg of the specific IP antibody or normal IgG (negative control) was added and incubated overnight at 4 °C. Subsequently, 10 μl of protein A beads were added and incubated for 30–45 min at 4 °C. The beads were collected by centrifugation at 7,000 rpm for 5 min and washed three times with RIPA buffer. Immunoprecipitated proteins were eluted by boiling the beads in 50 μl of 1 × sample loading buffer for 10 min before SDS-PAGE analysis.

### Statistical analysis

Values are presented as the mean ± SEM from at least three independent experiments. Statistical significance was assessed using Student’s *t*-test, with a *p* value < 0.05 considered statistically significant.

## Results

### Higher CASK gene expression in lung tumors is correlated with the poor survival of adenocarcinoma patients

**Fig. 1:**
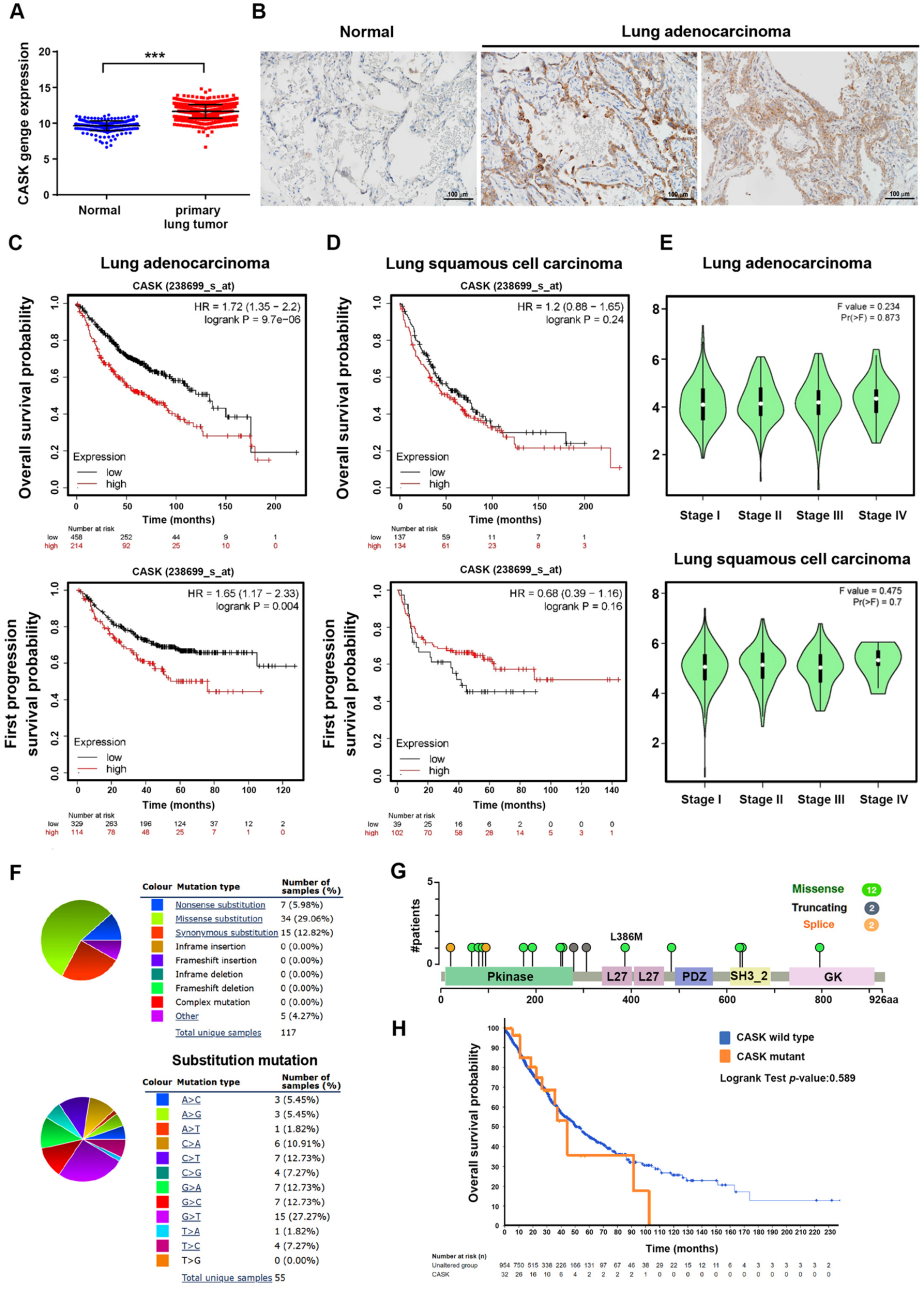
CASK expression and its association with lung cancer progression in patients with adenocarcinoma and squamous carcinoma. (**A**) The TCGA database was used to analyze CASK gene expression in patients with lung cancer. ***p < 0.001 indicates a significant difference between primary tumor tissues (n = 1,011) and normal tissues (n = 288). (**B**) Immunohistochemistry was performed to compare CASK protein expression in tumor and adjacent non-tumor tissues from patients with lung adenocarcinoma. Scale bars represent 100 μm. (**C, D**) The TCGA dataset was stratified by median CASK mRNA expression and analyzed using the Kaplan–Meier Plotter to evaluate the association of CASK expression in OS and FP survival in patients with lung adenocarcinoma (**C**) or lung squamous cell carcinoma (**D**). (**E**) GEPIA was used to analyze CASK gene expression in tumor tissues from patients at different pathological stages of lung adenocarcinoma and squamous cell carcinoma. (**F**) The COSMIC database was used to analyze the distribution of CASK mutation types (upper pie chart) and substitution mutations (lower pie chart). (**G, H**) The distribution of CASK mutations along the gene (**G**) and OS of patients harboring wild-type (n = 1018) or mutant CASK (n = 35) (**H**) were analyzed using the cBioPortal database

Previous studies have demonstrated that CASK is expressed across multiple tumor types, including colorectal, gastric, liver, prostate, and pancreatic cancers [6–10]. Elevated CASK expression is associated with poor prognosis and reduced survival in colorectal [6] and pancreatic cancers [8]. To investigate the role of CASK in NSCLC, we first interrogated bioinformatics data from the TCGA database. CASK mRNA levels were significantly higher in primary lung tumors (n = 1011) compared with normal lung tissues (n = 288) (Fig. 1A). Consistently, immunohistochemical analysis revealed elevated CASK protein expression in lung adenocarcinoma tissues relative to adjacent normal tissues (Fig. 1B). Kaplan–Meier analysis demonstrated that lung adenocarcinoma patients with high CASK expression exhibited reduced OS (n = 672) (Fig. 1C, upper panel) and FP survival (n = 874) (Fig. 1C, lower panel). In contrast, no significant association between CASK expression and OS or FP survival was observed in patients with lung squamous cell carcinoma (Fig. 1D). GEPIA analysis revealed that CASK gene expression was already elevated in stage I patients, with no significant differences observed between early and late stages of lung adenocarcinoma or lung squamous cell carcinoma (Fig. 1E). These results suggest that CASK upregulation occurs primarily at the early stages of NSCLC. Taken together, these findings support the potential of CASK as a biomarker and prognostic indicator in NSCLC. We further analyzed the mutation landscape of the CASK gene in lung cancers using the COSMIC database. Substitution mutations were the most common type observed. Specifically, the upper pie chart shows that 29.1% of mutations were missense, 12.8% were synonymous, and 6% were nonsense substitutions (Fig. 1F, upper panel). Among substitution mutations, G > T transversions were the most frequent, accounting for 27.3% (Fig. 1F, lower panel). Analysis via cBioPortal revealed that the majority of CASK mutations were located within the CaMK domain of the protein (Fig. 1G). Survival analysis using cBioPortal indicated no significant difference in OS between patients with wild-type CASK (n = 1018) and those with CASK mutations (n = 35) (Fig. 1H).

### Distinct gene expression profile in CASK knockdown PC9 and H1299 cells

**Fig. 2:**
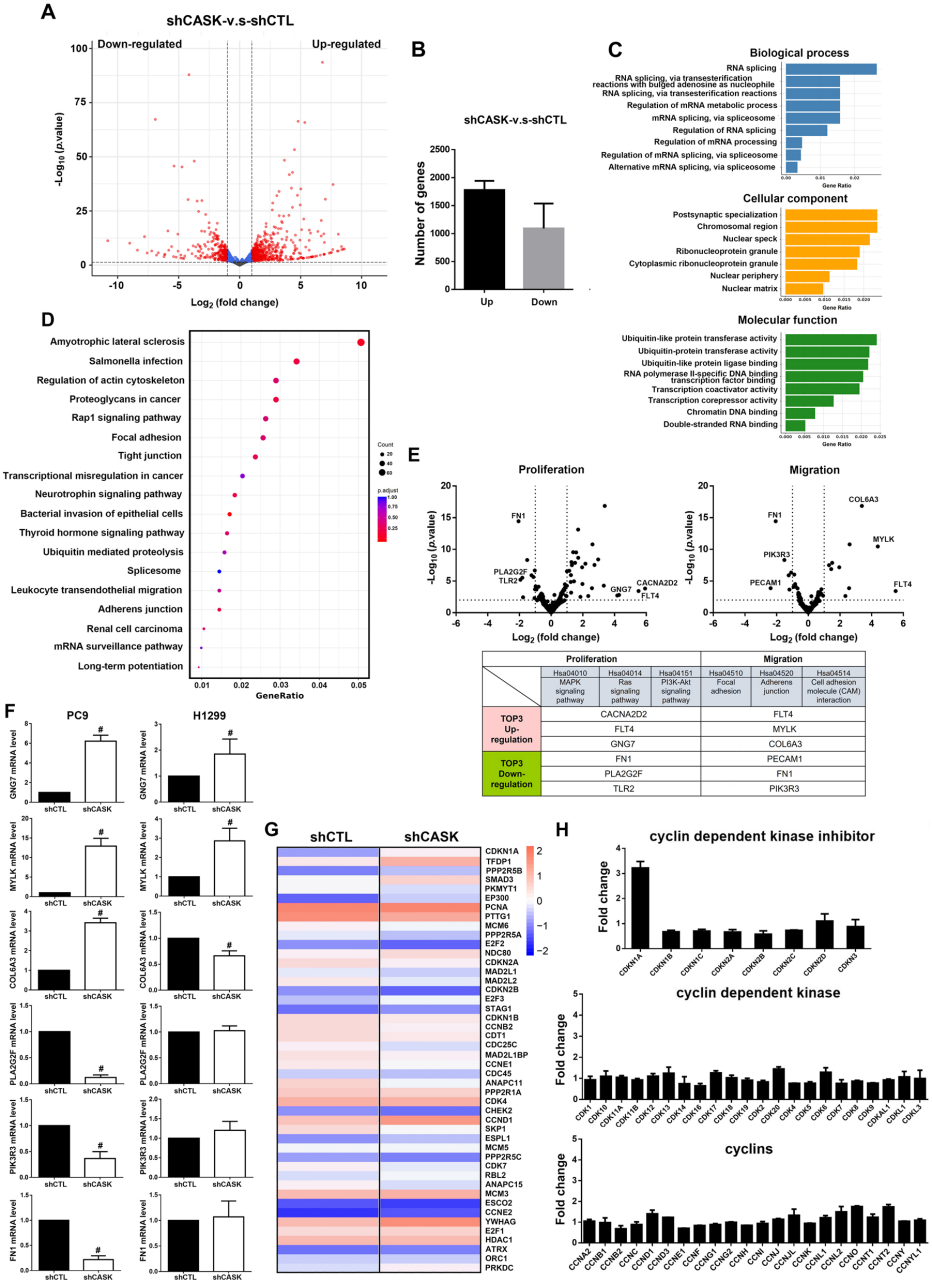
Assess the DEGs by RNA-Seq in PC9 cells. (**A**) Following CASK silencing, the distribution of upregulated and downregulated genes was shown in a volcano plot. A cutoff of |log₂ fold change|> 1 and *p* < 0.05 was applied. (**B**) Statistical analysis showed the number of DEGs upon CASK silencing. Fold-change thresholds of > 2 and < 0.5 were defined as the cutoffs for upregulated and downregulated genes, respectively. (**C**) Histograms of GO analysis showed significantly enriched GO terms in the biological process, cellular component, and molecular function categories. The gene ratio (x-axis) represented the proportion of genes of interest relative to the total DEGs within a given GO term. (**D**) KEGG pathway enrichment analysis showed enriched pathways following CASK silencing. Dot size indicated gene number, and color indicated the *p-value*. (**E**) Genes affected by CASK silencing and categorized into different biological processes, including cell proliferation and migration, were shown as volcano plots. (**F**) qPCR analysis was conducted to define the expression of the most-affected genes as listed in Fig. 2E in PC9 and H1299 cells. (**G**) A heat map showed relatively higher (red) and lower (blue) gene expression levels in shCTL and shCASK PC9 cells within the *Homo sapiens* cell cycle (hsa04110) pathway. (**H**) The effects of CASK silencing on the expression of CDK inhibitors, CDKs, and cyclins were shown

To investigate the role of CASK in NSCLC, we knocked down CASK in PC9 cells using lentiviral shRNA. RNA sequencing (RNA-Seq) was performed to identify differentially expressed genes (DEGs) between shCTL and shCASK cells. A total of 2,871 genes were significantly affected in shCASK cells compared with shCTL cells, with 1,780 genes (61.9%) upregulated and 1,092 genes (38%) downregulated, as shown in the volcano plot (Fig. 2A, B). GO enrichment analysis, encompassing biological process, cellular component, and molecular function categories, revealed that CASK regulates RNA splicing, mRNA metabolic processes, and mRNA processing in the biological process category; postsynaptic specialization, chromosomal region, and nuclear speck in the cellular component category; and ubiquitin-like protein transferase activity, ubiquitin-protein transferase activity, and ubiquitin-like protein ligase binding in the molecular function category (Fig. 2C). KEGG pathway analysis further indicated that CASK is involved in pathways regulating the actin cytoskeleton, focal adhesion, tight junctions, and adherens junctions (Fig. 2D). We next focused on CASK-regulated genes associated with cell proliferation and migration. Proliferation-related pathways included MAPK (hsa04010), Ras (hsa04014), and PI3K-AKT (hsa04151), while migration-related pathways included focal adhesion (hsa04510), adherens junction (hsa04520), and cell adhesion molecule interactions (hsa04514). The top three upregulated and downregulated genes in both pathways were displayed in the volcano plot (Fig. 2E). To validate the changes in the expression of these most affected genes, we performed qPCR analysis of some genes in PC9 and H1299 cells. The results showed that shCASK increased the expression of GNG7, MYLK, and COL6A3, but decreased the expression of PLA2G2F, PIK3R3, and FN1 in PC9 cells (Fig. 2F). These changes were consistent to those detected by RNA-Seq (Fig. 2E). In H1299 cells, shCASK increased gene expression of GNG7 and MYLK, but not COL6A3. Of note, the three genes downregulated in shCASK PC9 cells were not changed in H1299 cells (Fig. 2F). Moreover, heatmap analysis of the Homo sapiens cell cycle pathway (hsa04110) highlighted the top 50 significantly up- and downregulated gene expression in shCASK versus shCTL PC9 cells (Fig. 2G). Analysis of cell cycle regulators revealed that p21 gene (i.e., CDKN1A) expression was approximately threefold higher in shCASK cells compared with shCTL cells (Fig. 2H, upper panel). In contrast, expression levels of CDKs (Fig. 2H, middle panel) and cyclins (CCNs; Fig. 2H, lower panel) were not significantly altered. These results suggest that CASK selectively modulates p21 expression without broadly affecting other core cell cycle regulators.

### CASK silencing inhibits lung cancer cell growth and inhibits the progression to the S phase

**Fig. 3:**
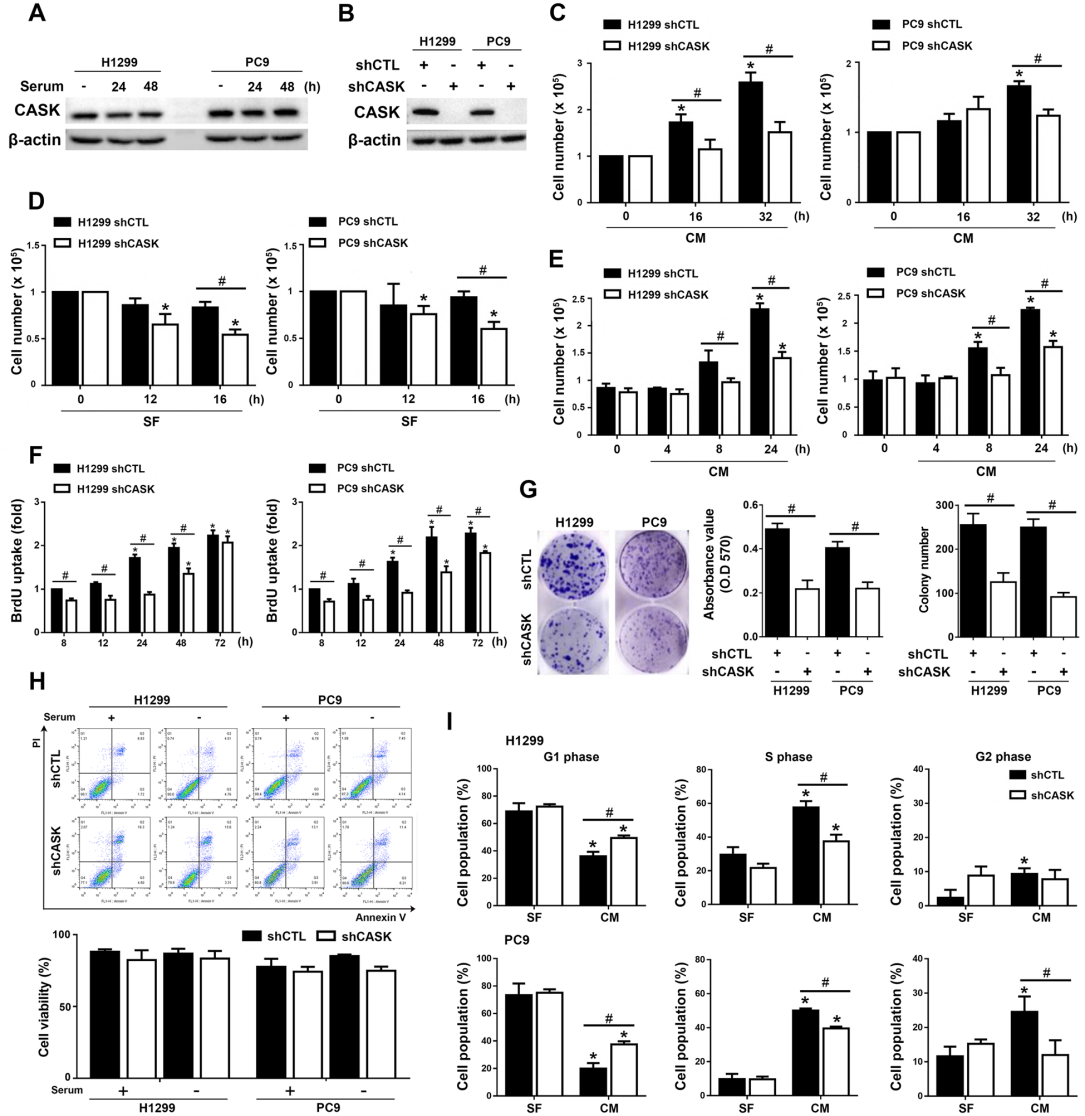
CASK silencing reduces cell growth in H1299 and PC9 cells. (**A**) Cells were cultured in SF medium and then transferred to CM. CASK protein levels were determined by immunoblotting. (**B**) CASK knockdown efficiency was determined by immunoblotting. (**C–E**) A Trypan blue exclusion assay was performed to count live, unstained cells at the indicated times after continuous culture in CM (**C**), continuous culture in SF medium (**D**), or overnight serum starvation followed by transfer to CM (**E**). (**F**) A BrdU assay was used to determine cell proliferation in H1299 and PC9 cells after culture in CM for the indicated times. (**G**) A colony formation assay was conducted by counting colony numbers and measuring the absorbance after crystal violet staining. (**H**) Cell viability was assessed by Annexin V/PI staining after culture in SF medium or CM for 24 h. (**I**) PI staining followed by flow cytometry was conducted to assess serum-induced cell cycle progression at 24 h in H1299 and PC9 cells. The G1, S, and G2/M phase distributions were analyzed using ModFit LT™ software. Data are presented as mean ± S.E.M. from at least three independent experiments. *p < 0.05 indicates significant effects of serum and SF conditions on cell growth (**C**–**F**) and cell cycle progression (**I**). #*p* < 0.05 indicates significant effects of CASK silencing on cell number (**C**–**E**), BrdU incorportation (**F**), colony formation (**G**), and cell cycle regulation (**I**)

To investigate the role of CASK in NSCLC cell growth, we first examined CASK protein levels in cells cultured in complete medium (CM) or serum-free (SF) medium. CASK expression in both EGFR wild-type H1299 and EGFR exon 19 deletion PC9 cells remained unchanged under CM or SF conditions (Fig. 3A). Next, we knocked down CASK in H1299 and PC9 cells using lentiviral shRNA (Fig. 3B). Cell counting and colony formation assays demonstrated that CASK knockdown significantly inhibited proliferation in both cell lines. Cells were cultured under three conditions: continuously in CM, continuously in SF medium, or in SF medium overnight followed by serum re-addition for the indicated times. H1299 cells exhibited faster growth than PC9 cells in CM. In all conditions, shCASK cells showed reduced cell numbers compared with controls, whether cultured in CM for 32 h (Fig. 3C), in SF medium for 16 h (Fig. 3D), or in SF medium followed by serum re-addition for 24 h (Fig. 3E). Consistently, BrdU incorporation, a measure of DNA synthesis in S phase, was decreased in shCASK H1299 cells cultured from 8 to 48 h in CM. The BrdU incorporation was similarly inhibited in shCASK PC9 cells (Fig. 3F). Colony formation assays further confirmed that loss of CASK reduced colony numbers in both H1299 and PC9 cells (Fig. 3G). To assess whether changes in cell viability contributed to altered cell numbers, we measured apoptosis using Annexin V/PI staining. The percentages of viable cells were comparable between shCTL and shCASK cells, regardless of CM or SF culture for 24 h (Fig. 3H). Analysis of cell cycle progression revealed that, although both shCTL and shCASK cells exited G1 and entered S phase following 24 h of serum stimulation, shCASK reduced the proportion of cells in S phase, indicating delayed cell cycle progression in both cell lines (Fig. 3I). Collectively, these results indicate that CASK promotes NSCLC cell proliferation without affecting cell viability.

### CASK silencing inhibits the cell cycle via upregulation of p21 expression

**Fig. 4:**
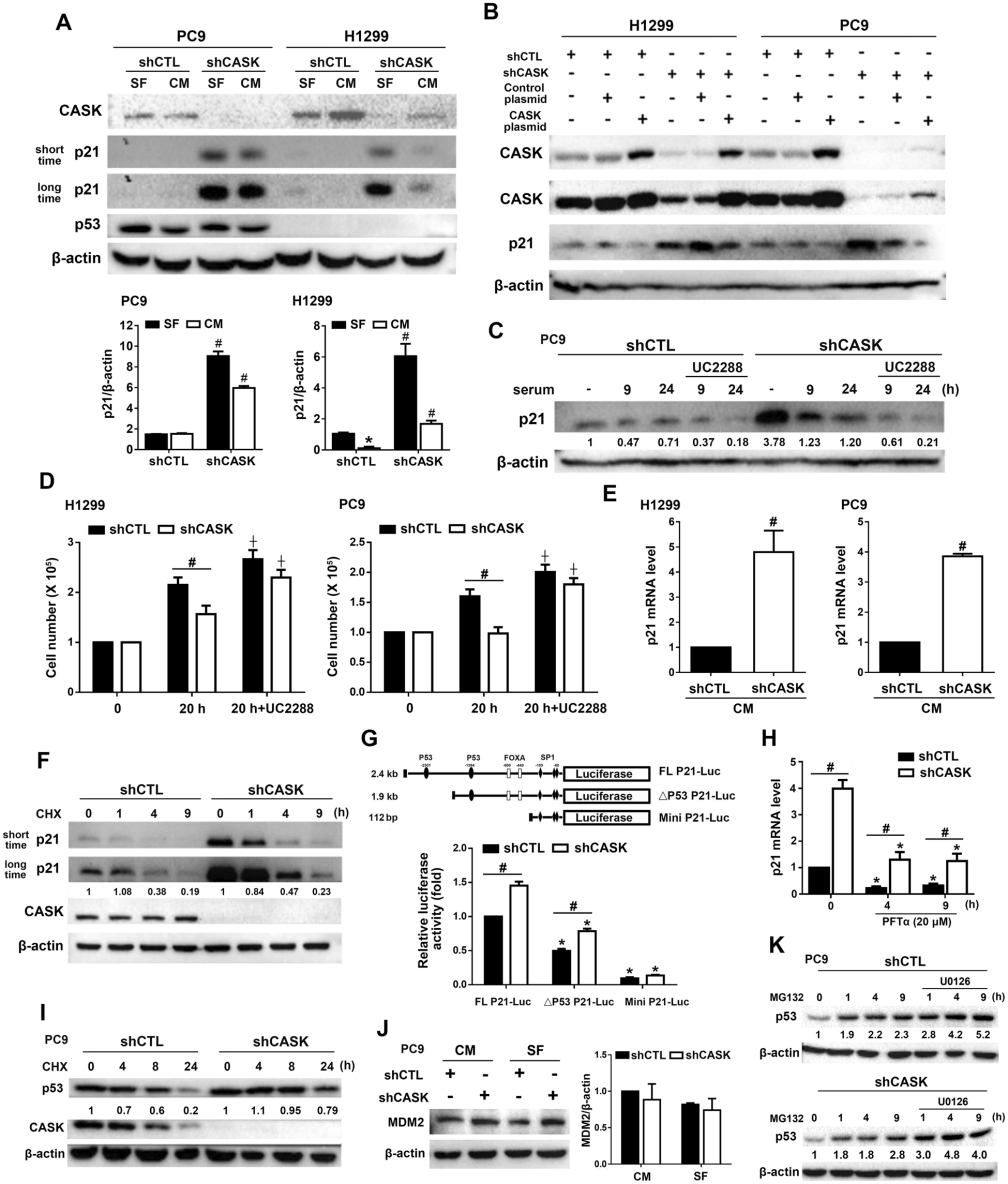
CASK silencing increases p21 expression in H1299 and PC9 cells. (**A**) H1299 and PC9 cells were cultured in either SF medium or CM, and the protein levels of p21 and p53 were determined. (**B**) CASK was overexpressed in H1299 and PC9 cells, and p21 protein expression was determined by Western blotting. (**C, D**) Cells were first cultured in SF medium, and then incubated in CM in the absence or presence of UC2288 (10 µM) for the indicated times. p21 protein levels (**C**) and cell growth (**D**) were determined. (**E**) RT-PCR was conducted to analyze p21 mRNA levels in H1299 and PC9 shCTL and shCASK cells. (**F**) Cells were treated with CHX (10 μg/ml) for the indicated times to assess the protein stability of p21 by Western blotting. (**G**) p21 promoter assay was assessed to determine the luciferase activity of p21 in cells transfected with full-length (FL) p21 construct, p53 binding site-deleted construct and minimal p21 construct. (**H**) qPCR analysis was performed to determine the p21 mRNA levels under PFTα (20 µM) treatment for 4 and 9 h in PC9 cells. (**I**) Cells were treated with CHX (10 μg/ml) for the indicated times to assess the protein stability of p53 by Western blotting. (**J**) Cells were cultured in either SF medium or CM conditions, and the protein level of MDM2 was determined by Western blotting. (**K**) Cells were pre-treated with U0126 (10 µM) for 30 min, then treated with MG132 (10 µM) for the indicated time. The protein level of p53 was determined by Western blotting. Data are presented as mean ± S.E.M. from at least three independent experiments. *p < 0.05 indicates significant effects of serum and PFTα on p21 protein expression (**A**) and p21 mRNA expression (**H**), respectively, as well as the significant effects of cells transfected with different plasmid constructs on p21 luciferase activity (**G**). #*p* < 0.05 indicates significant effects of CASK silencing on p21 protein (A), cell growth (**D**), mRNA expression (**E**, **H**), and p21 luciferase activity (**G**). ⨥ < 0.05 indicates a significant effect of UC2288 on serum-induced cell growth in both shCTL and shCASK cells (**D**)

To investigate the role of CASK in cell cycle regulation, we first examined p21 protein expression. In both PC9 and H1299 cells, cultured in either SF medium or CM, p21 protein levels were elevated in shCASK cells compared with controls. Of note, such an increase was more obvious in SF conditions than in CM, especially in H1299 cells (Fig. 4A). Conversely, overexpression of CASK in PC9 and H1299 cells reduced p21 protein levels (Fig. 4B). To determine the functional role of p21 in CASK-regulated cell cycle progression, we used the pharmacological p21 inhibitor UC2288, which decreases p21 mRNA and protein levels via a p53-independent, yet unidentified mechanism [29]. When cells were switched from SF medium to CM, p21 protein levels were decreased in both shCTL and shCASK PC9 cells, and UC2288 treatment further decreased this effect (Fig. 4C). After demonstrating that UC2288 downregulates p21, we used it to evaluate whether p21 is involved in the shCASK-induced inhibition of cell growth in H1299 and PC9 cells. After 20 h of serum stimulation, UC2288 enhanced cell growth in shCTL cells and significantly reversed the growth-inhibiting effects of shCASK (Fig. 4D). This finding indicates that p21 upregulation mediates the growth-suppressive effect of shCASK.

Subsequently, we examined p21 expression to explore the underlying mechanism. First, we found that CASK silencing upregulated p21 gene expression in both H1299 and PC9 cells (Fig. 4E). Second, we assessed p21 protein stability using cycloheximide (CHX) treatment. The results showed that CASK silencing did not affect p21 stability in PC9 cells (Fig. 4F). Next, to determine whether p53 is involved in shCASK-mediated transcriptional upregulation of p21, we performed a p21 promoter assay and treated cells with the p53 inhibitor PFTα (20 μM). We observed that CASK silencing enhanced p21 promoter activity in cells transfected with the full-length construct. Although deletion of the p53-binding site markedly reduced luciferase activity in both shCTL and shCASK PC9 cells compared with the intact promoter, CASK knockdown still increased p21 activity to a comparable degree in both the full-length and p53-deleted constructs. In contrast, the activity of the minimal p21 promoter exhibited a pronounced suppression of activity under the same conditions (Fig. 4G). Comparable effects were observed upon p53 inhibition. Although treatment with PFTα reduced p21 mRNA levels in both shCTL and shCASK PC9 cells, CASK silencing nevertheless elevated p21 expression under PFTα exposure (Fig. 4H). Collectively, these findings indicate that CASK knockdown–mediated induction of p21 transcription may proceed through p53-independent mechanisms.

We next examined whether CASK regulates p53 expression. Under both SF and CM conditions, p53 protein levels remained unchanged in PC9 shCASK cells (Fig. 4A, I). However, CHX treatment revealed that CASK silencing enhanced p53 protein stability in PC9 cells (Fig. 4I). MDM2 is a well-established E3 ubiquitin ligase that mediates p53 degradation, and its activity is reinforced by ERK-dependent phosphorylation, which stabilizes MDM2 and augments its ubiquitination capacity [30, 31]. To determine whether CASK modulates MDM2-dependent proteasomal degradation of p53, we assessed MDM2 protein levels. CASK silencing did not affect MDM2 expression under both CM and SF conditions (Fig. 4J). Treatment with the proteasome inhibitor MG132 elevated p53 protein levels to a similar extent in shCTL and shCASK PC9 cells (Fig. 4K). Moreover, MG132-induced stabilization of p53 was further augmented by ERK inhibition, with comparable fold increases observed in both shCTL and shCASK cells (Fig. 4K). Together, these findings support a role for ERK–MDM2 signaling in regulating p53 turnover at the resting state of PC9 cells, while the stabilization of p53 protein induced by CASK silencing appears to occur independently of the ERK–MDM2-p53 axis.

### CASK silencing affects cell cycle regulators and mitosis

**Fig. 5:**
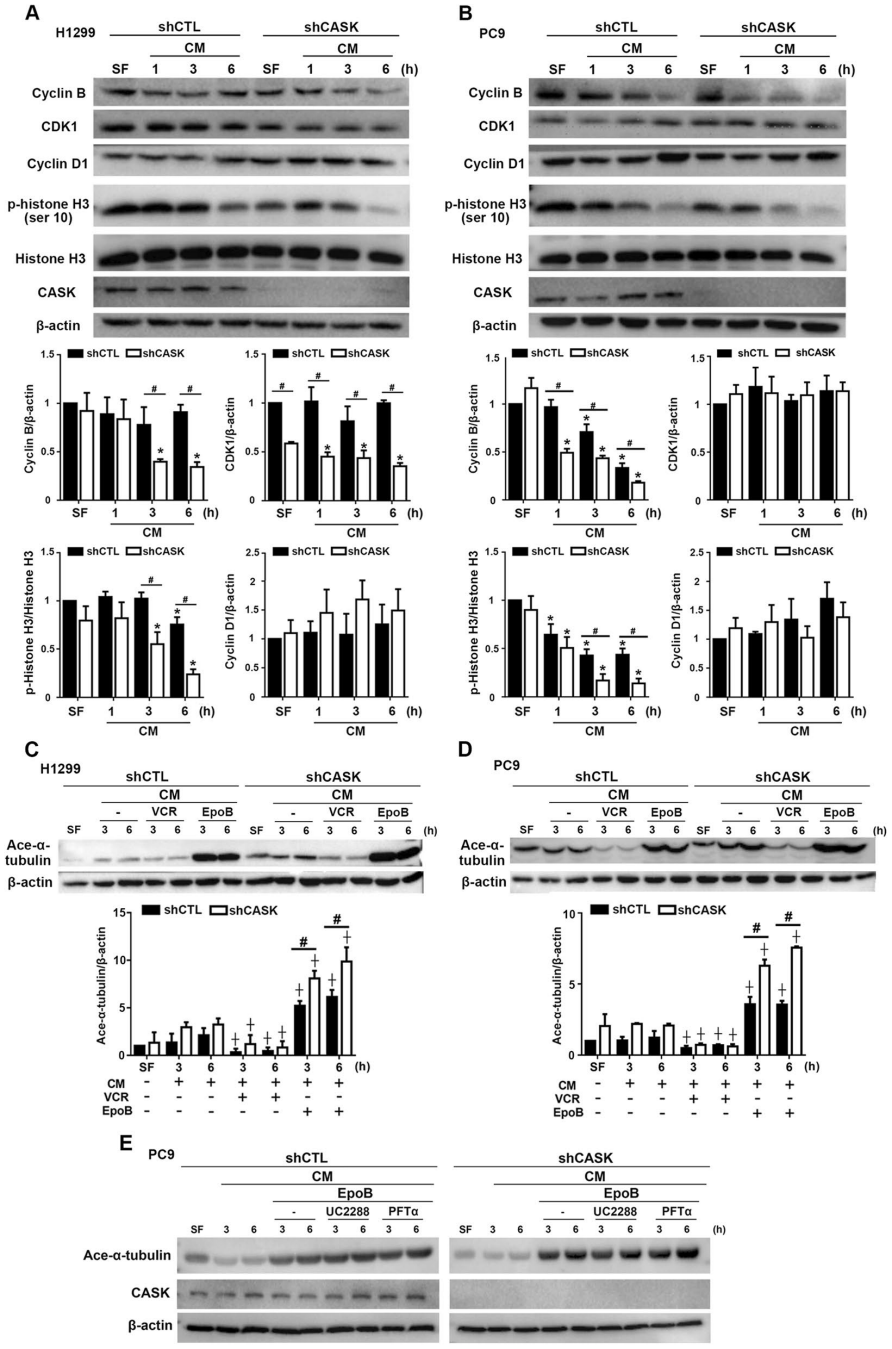
CASK silencing inhibits cell cycle regulators and promotes tubulin stabilization in H1299 and PC9 cells. Serum-starved H1299 (**A, C**) and PC9 (**B, D, E**) cells were incubated in CM for the indicated times. Protein levels of cyclin B, CDK1, cyclin D1, and phosphorylated histone H3 were determined by Western blotting (**A, B**). In some experiments, cells were treated with vincristine (1 μM), EpoB (50 nM), UC2288 (10 μM), or PFT-α (10 μM), after which acetylated α-tubulin levels were determined (**C–E**). Data are presented as the mean ± S.E.M. from at least three independent experiments. *p < 0.05 indicates a significant effect of serum stimulation on protein expression. #*p* < 0.05 indicates a significant effect of CASK silencing on protein expression. ⨥*p* < 0.05 indicates significant effects of vincristine and EpoB on acetylated α-tubulin levels

To further investigate the role of CASK in cell cycle regulation, we examined the expression of key cell cycle regulators. In H1299 cells, CASK silencing reduced cyclin B and CDK1 expression after serum stimulation, while cyclin D1 levels remained unchanged. Phosphorylation of histone H3 at Ser10 (p-H3) is a marker of mitosis [32], and p-H3 levels were decreased in shCASK H1299 cells (Fig. 5A). Similarly, in PC9 cells, CASK knockdown reduced cyclin B and p-H3 levels, but did not affect CDK1 or cyclin D1 expression (Fig. 5B). Given that microtubules, composed of α/β-tubulin heterodimers, are critical for chromosome movement and segregation during mitosis, we next assessed the effect of CASK on tubulin dynamics. Acetylation of α-tubulin promotes microtubule stabilization and G2/M cell cycle arrest [33]. Cells were treated with vincristine, which inhibits tubulin polymerization, or EpoB, which promotes it. Vincristine reduced acetylated α-tubulin levels in both cell lines, and this effect was not influenced by CASK silencing. In contrast, EpoB induced α-tubulin acetylation, and this induction was further enhanced in CASK-silenced H1299 (Fig. 5C) and PC9 cells (Fig. 5D). Notably, inhibition of p53 or p21 did not affect EpoB-induced α-tubulin acetylation (Fig. 5E). These findings suggest that, in addition to regulating cell cycle proteins, CASK modulates mitosis by influencing α-tubulin acetylation and microtubule dynamics.

### CASK silencing upregulates EGFR expression via gene transcription and protein stabilization

**Fig. 6:**
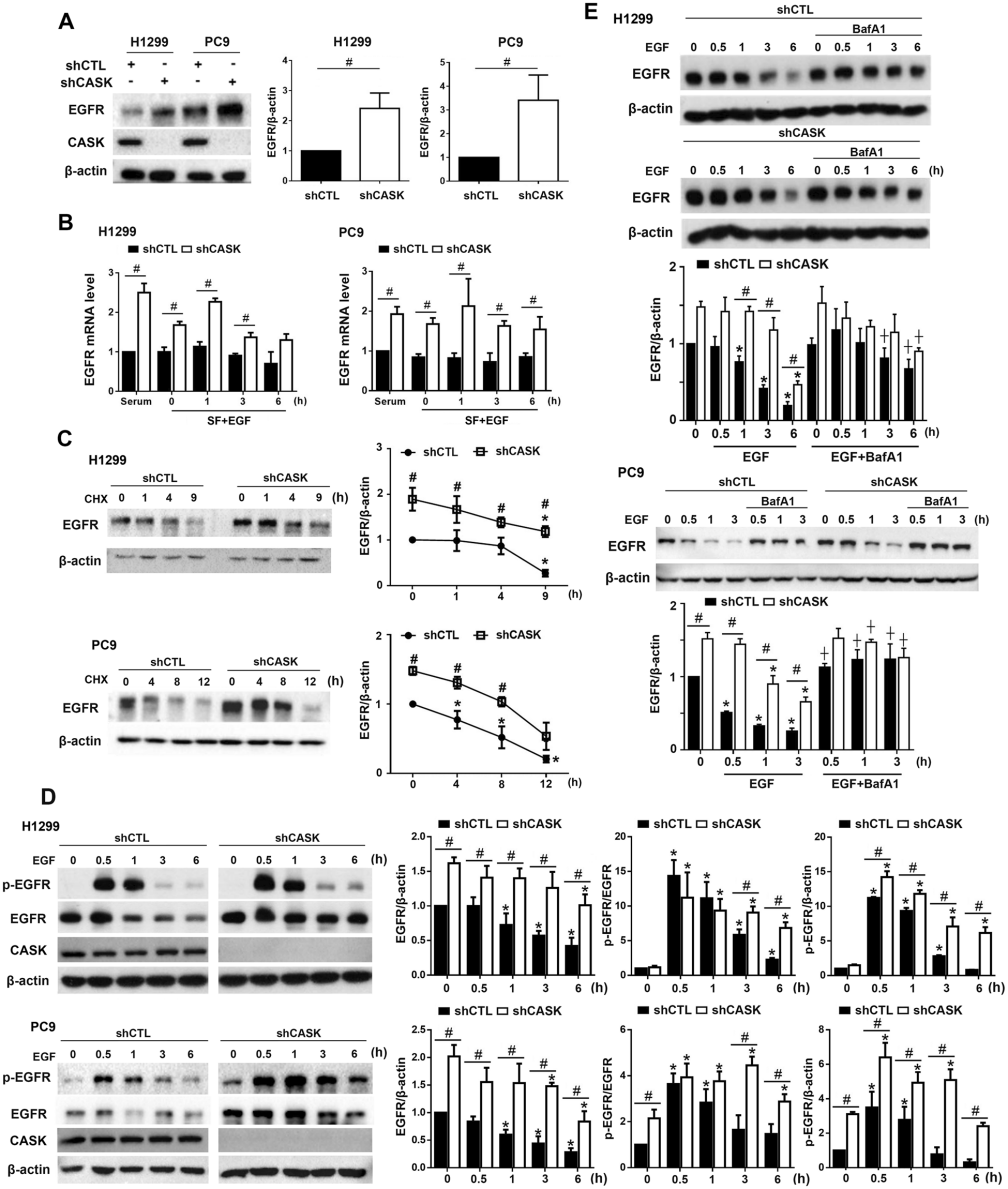
CASK silencing upregulates EGFR expression and activation in H1299 and PC9 cells. (**A**) The effect of CASK silencing on EGFR protein expression in cells cultured in complete medium (CM) was determined by Western blotting. (**B**) The effect of CASK silencing on EGFR mRNA expression before and after EGF treatment was assessed by RT–PCR. (**C**) CHX (10 μg/ml) was used to assess the effect of CASK silencing on EGFR protein stability under resting conditions. (**D**) The effects of CASK silencing on EGFR activation and degradation following EGF treatment (50 ng/ml) for the indicated times were determined by Western blotting. (**E**) Cells were pretreated with BafA1 (100 nM) for 1 h and then treated with EGF (50 ng/ml) for the indicated times, after which EGFR protein levels were determined by Western blotting. Data are presented as the mean ± S.E.M. from at least three independent experiments. *p < 0.05 indicates significant effects of CHX on EGFR protein stability (**C**) and significant effects of EGF on EGFR activation (**D**) and degradation (**E**). #*p* < 0.05 indicates significant effects of CASK silencing on EGFR protein (**A**) and mRNA (**B**) expression, protein stability (**C**), EGFR activation (**D**), and degradation (**E**). ⨥*p* < 0.05 indicates significant effects of BafA1 on EGF-induced EGFR degradation

CASK has been shown to regulate EGFR function in *C. elegans* by mediating the localization and recruitment of EGFR to the basolateral plasma membrane [14, 15]. In addition, our previous study in microglial cells showed the effect of CASK silencing to increase EGFR expression [34]. Therefore, we addressed the role of CASK in EGFR activation in NSCLC. First, we found an enhanced EGFR protein expression upon CASK silencing in H1299 and PC9 cells (Fig. 6A). After EGF treatment for the indicated times, CASK-silencing cells can still exhibit a higher mRNA level of EGFR compared to shCTL cells (Fig. 6B). These data suggest that CASK exerts a negative role in EGFR gene expression. Besides regulating gene expression, we wonder if CASK might also regulate EGFR protein stability. To address this issue, we treated cells with CHX (10 μg/ml). We found that the half-life of EGFR protein was 4.4 $\pm$ 1.2 h in shCTL H1299 cells, while it was longer than 9 h in shCASK H1299 cells (Fig. 6C, upper panel). Similarly, the EGFR half-life values were 4.62 $\pm$ 0.9 h in shCTL cells and higher than 9 h in shCASK PC9 cells (Fig. 6C, lower panel). These findings indicate that CASK also plays a role in decreasing EGFR protein stability. After observing the role of CASK in the regulation of EGFR expression, we were interested in understanding EGFR activity. We treated EGF in pre-starved cells. Results showed that exogenous EGF treatment can rapidly induce EGFR phosphorylation, which was followed by a significant EGFR downregulation in shCTL H1299 and PC9 cells. In shCASK cells, the EGFR phosphorylation was prolonged, and the receptor activation-induced EGFR downregulation was attenuated (Fig. 6D). The half-life values of EGFR under EGF stimulation were 3.9 $\pm$ 0.9 h and 2.6 $\pm$ 0.9 h in shCTL H1299 and PC9 cells, respectively, and were more than 6 h in shCASK H1299 and PC9 cells (Fig. 6D). Along with EGFR downregulation after EGF stimulation, CASK protein levels were not affected (Fig. 6D). Again, these findings under EGF stimulation confirm the negative role of CASK in EGFR stability. To understand how to regulate EGFR degradation, we determined the lysosomal pathway by treating cells with lysosome inhibitor BafA1. We found that BafA1 can significantly reverse EGF-induced rapid EGFR degradation starting at 1 h in shCTL H1299 cells and 0.5 h in shCTL PC9 cells. However, this apparent effect of BafA1 was not detected in shCASK cells until 6 h in H1299 cells and 1 h in PC9 cells (Fig. 6E). These findings suggest that CASK is involved in mediating lysosomal degradation of EGFR, and CASK silencing delays such degradation.

### CASK co-localizes with EGFR, and CASK silencing decreases EGFR entry into the late endosome

**Fig. 7:**
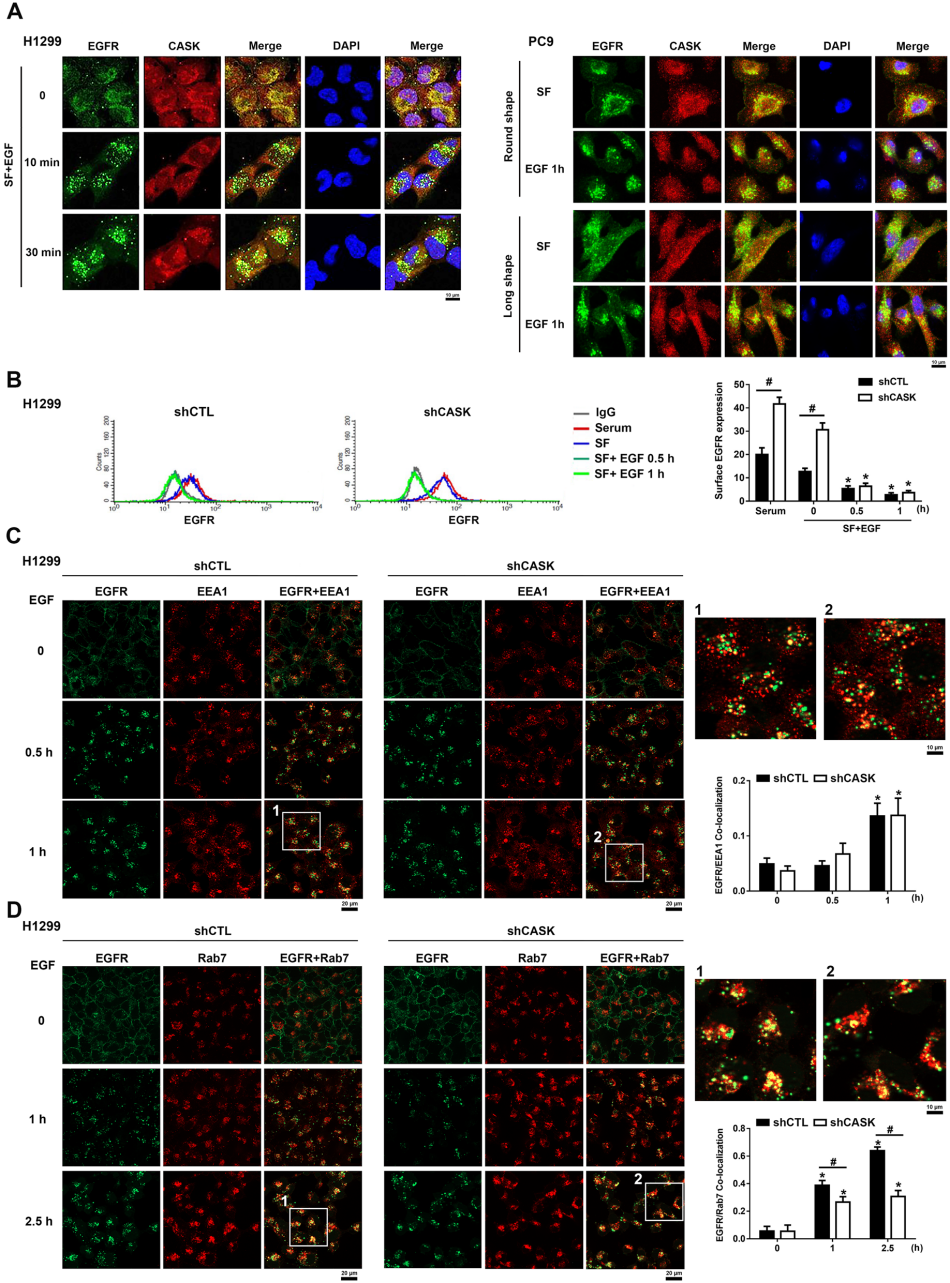
CASK silencing delays EGFR trafficking into the late endosome and increases EGFR level at the plasma membrane. (**A**) Immunofluorescence staining was used to determine the subcellular localization of CASK and EGFR in H1299 and PC9 cells following EGF stimulation (50 ng/ml) for the indicated times. Scale bars represent 10 μm. (**B**) The effect of CASK silencing on EGFR plasma membrane expression in H1299 cells was analyzed by flow cytometry. (**C, D**) The effects of CASK silencing on EGFR colocalization with EEA1 (**C**) and Rab7 (**D**) in PC9 cells were assessed by immunofluorescence staining. Scale bars represent 20 μm. Enlarged views of selected regions are shown on the right. Data are presented as the mean ± S.E.M. from at least three independent experiments. *p < 0.05 indicates significant effects of EGF on EGFR plasma membrane expression (**B**) and on the colocalization of EGFR with EEA1 (**C**) and Rab7 (**D**). #*p* < 0.05 indicates significant effects of CASK silencing on EGFR plasma membrane expression (**B**) and on the colocalization of EGFR with Rab7 (**D**)

Next, we were interested in how CASK regulated EGFR trafficking in lung cancer cells. There are two cell morphologies of PC9 cells, i.e., round and long shapes. Immunofluorescence staining showed that CASK was predominantly localized in the cytosol and co-localized with EGFR in H1299 and PC9 cells at the resting state. Upon EGF stimulation for 10 min in H1299 cells, plasma membrane EGFR was internalized into the cytosol and accumulated as punctate structures in perinuclear regions. At this time point, cytosolic co-localization with partner proteins was transiently reduced. In contrast, in PC9 cells stimulated with EGF for 1 h, CASK–EGFR colocalization remained unchanged (Fig. 7A).

To understand the EGFR level at the plasma membrane, we determined the fluorescence of 488-conjugated EGFR by flow cytometry. We found that the plasma membrane expression level of EGFR was enhanced in H1299 shCASK cells both in SF medium and CM conditions. Moreover, a rapid loss of EGFR level at the plasma membrane after EGF treatment for 0.5 and 1 h was observed, indicating the receptor internalization. However, such internalization rate was the same in shCTL and shCASK cells (Fig. 7B), suggesting that CASK silencing does not affect EGF-induced EGFR internalization. To further understand the role of CASK in regulating the EGF-induced EGFR trafficking pathway, we assessed the co-localization of EGFR with EEA1 and Rab7, respectively, by immunofluorescent staining to examine whether CASK affects EGFR entering into early- and late-endosomes. After EGF treatment for 0.5 and 1 h, the co-localization of EGFR and EEA1 was not changed by CASK silencing (Fig. 7C), while the co-localization of EGFR and Rab7 was decreased in CASK-silenced cells under EGF treatment for 2.5 h (Fig. 7D). Altogether, our data suggest that CASK silencing does not affect EGFR trafficking to early endosomes, but delays EGFR trafficking to late endosomes.

### CASK silencing increases EGFR activation and TGF-α expression via ERK, AKT, and p53 pathways

**Fig. 8:**
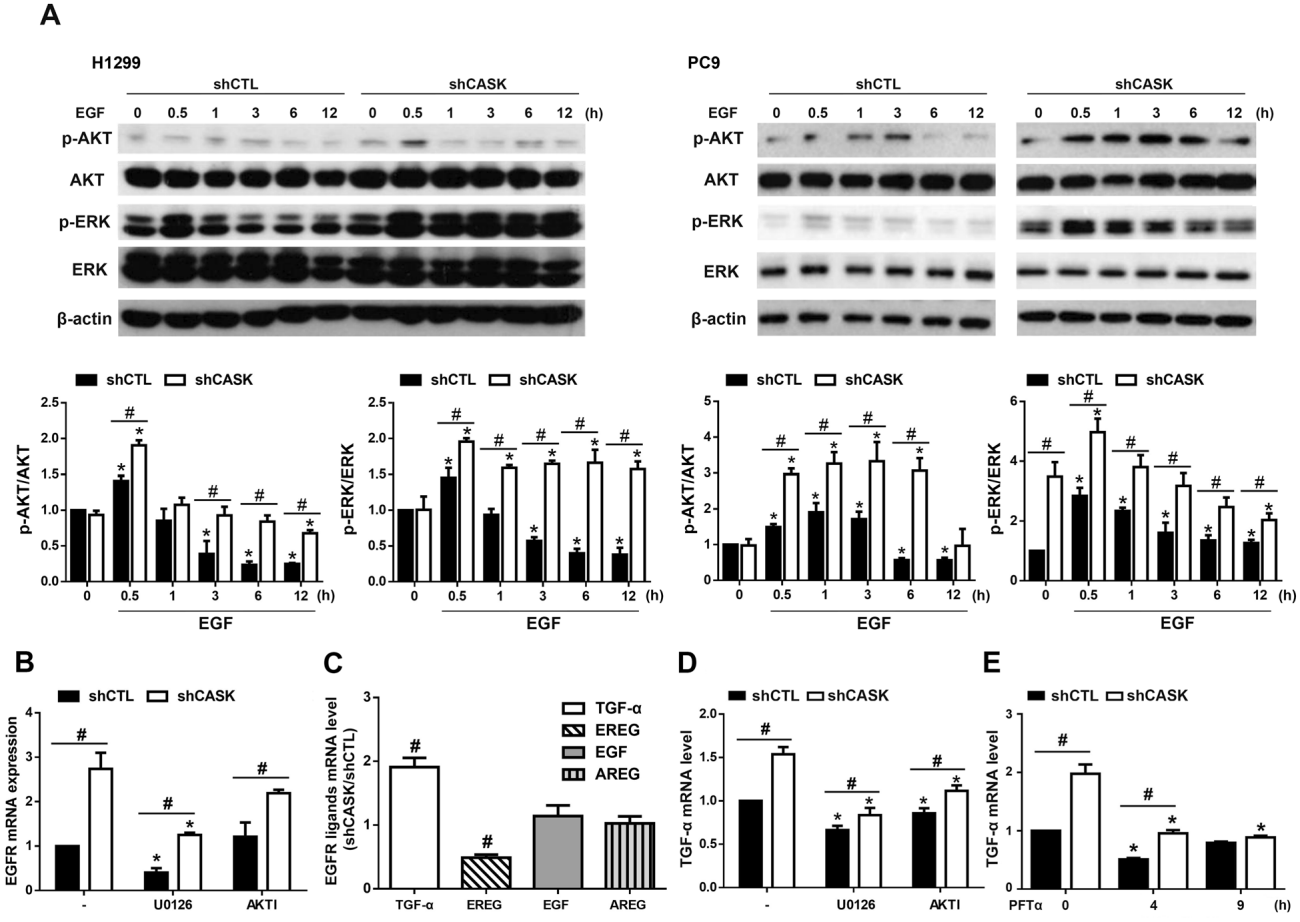
CASK silencing increases EGFR downstream ERK and AKT signaling and endogenous TGF-α expression. (**A**) Serum-starved H1299 and PC9 cells were treated with EGF (50 ng/ml) for the indicated times, and ERK and AKT phosphorylation were assessed. (**B**) PC9 cells cultured in CM were treated with U0126 (10 μM) or AKTI (10 μM) for 3 h, after which EGFR mRNA expression was analyzed by RT–PCR. (**C**) The mRNA levels of EGFR ligands in PC9 cells cultured in CM under resting conditions were determined by RT–PCR. (**D, E**) TGF-α mRNA levels in PC9 cells were determined by RT–PCR following treatment with U0126 or AKTI for 3 h (**D**) or with PFTα (10 μM) for 4 or 9 h (**E**). Data are presented as the mean ± S.E.M. from at least three independent experiments. *p < 0.05 indicates significant effects of EGF on ERK and AKT phosphorylation (**A**), and the significant effects of U0126, PFTα, and AKTI on EGFR and TGF-α mRNA expression (**B**, **C**, **D**, **E**). #*p* < 0.05 indicates significant effects of CASK silencing on ERK and AKT phosphorylation as well as TGF-α, EREG, and EGFR mRNA expression

After observing the increased EGFR expression and activation in shCASK cells (Fig. 6D), we wondered if the major downstream signaling pathways of EGFR, i.e., AKT and ERK, are also affected. Concomitantly, we found that both shCASK H1299 and PC9 cells exhibited higher levels of AKT and ERK phosphorylation after EGF treatment as compared to those in shCTL cells. Of note, CASK silencing in PC9, but not in H1299 cells, demonstrated a higher basal level of ERK phosphorylation (Fig. 8A). This cell type-specific effect on ERK activation is associated with the constitutive EGFR activation that was only seen in CASK silencing PC9 but not in H1299 cells (Fig. 6D). Next, we found that the increased EGFR gene expression in PC9 cells was attenuated by ERK inhibitor U0126 and AKTI (Fig. 8B). Moreover, besides upregulation of EGFR, we wondered if endogenous EGFR ligands might be affected by CASK silencing, and in turn contribute to EGFR activation. We detected several EGFR ligand mRNA expressions, including TGF-α, epiregulin (EREG), EGF, and amphiregulin (AREG), in PC9 cells. EREG and AREG are critical EGFR ligands that drive lung cancer progression and therapeutic resistance [35, 36]. Results showed that CASK silencing increased TGF-α gene expression, decreased EREG gene expression, and had no effects on EGF and AREG mRNA expressions (Fig. 8C). Accordingly, we deciphered the molecular mechanism for enhanced TGF-α gene expression in shCASK PC9 cells. Because the TGF-α gene is a direct target for p53-mediated transcriptional activation [37], we determined the roles of ERK, AKT, and p53. We found that CASK silencing-induced TGF-α mRNA expression at the resting state was inhibited by treatment with U0126 and AKTI for 3 h (Fig. 8D), as well as by PFTα treatment within 9 h (Fig. 8E). All these findings reveal the existence of a positive loop between endogenous TGF-α expression and constitutive EGFR activation in shCASK PC9 cells, and ERK, AKT, and p53 mediate this event.

### CASK silencing increases p21 expression via EGFR-AKT/ERK signaling

**Fig. 9:**
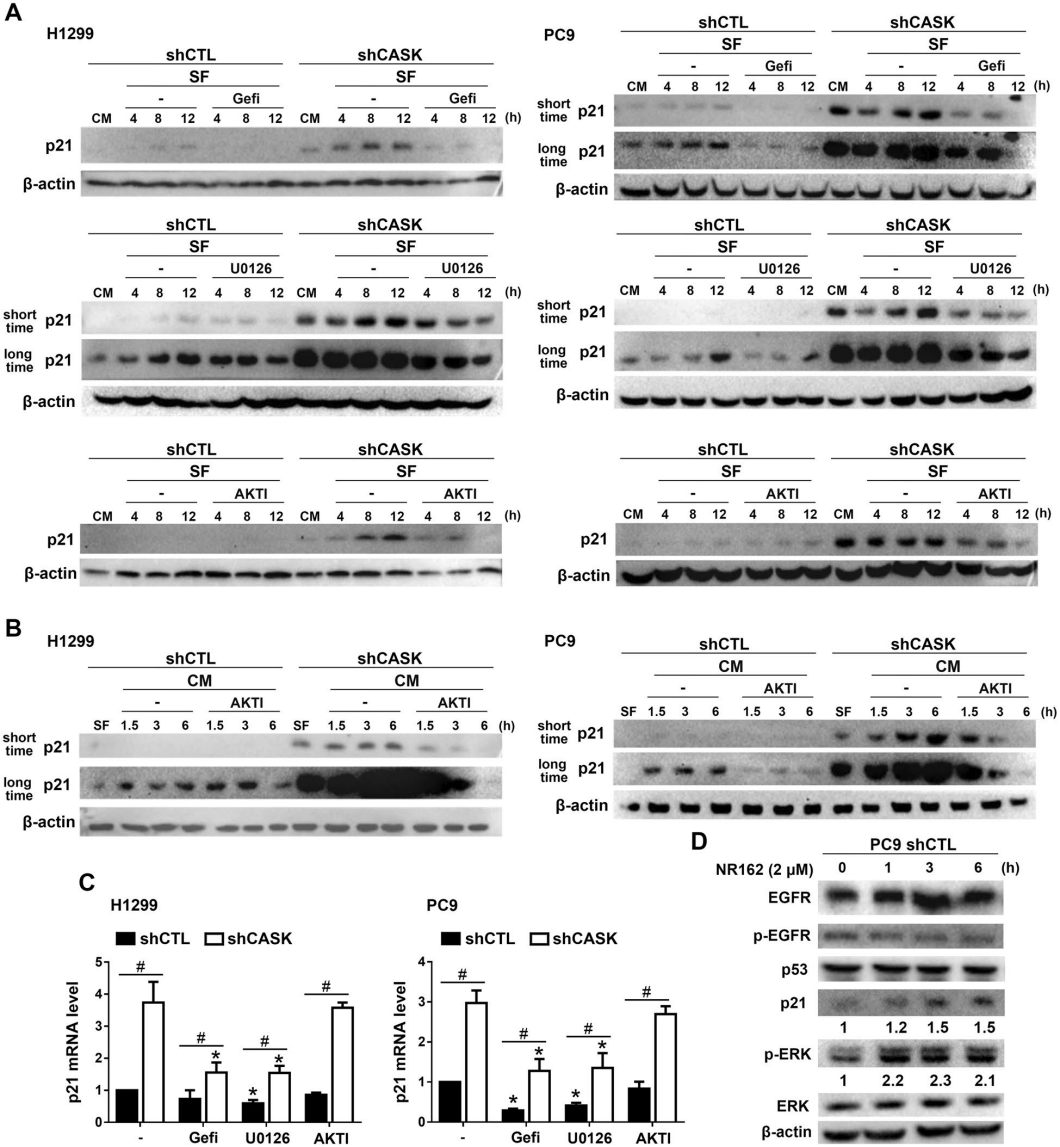
CASK silencing-induced p21 expression is dependent on EGFR signals. (**A**) H1299 and PC9 cells were cultured in CM overnight and then switched to SF medium in the presence or absence of gefitinib (10 μM), U0126 (10 μM), or AKTI (10 μM). (**B**) H1299 and PC9 cells were cultured in SF medium overnight and then transferred to CM in the presence or absence of AKTI (10 μM). After incubation for the indicated times, p21 protein levels were determined by immunoblotting. (**C**) Cells cultured in CM were treated with gefitinib, U0126, or AKTI for 8 h, after which p21 mRNA levels were analyzed by RT–PCR. (**D**) Cells were treated with NR162 (2 μM) for the indicated time. The protein levels of p-EGFR, EGFR, p21, p53, p-ERK, and ERK were determined by Western blotting. Data are presented as the mean ± S.E.M. from at least three independent experiments. *p < 0.05 indicates significant effects of gefitinib and U0126 on p21 mRNA expression. #*p* < 0.05 indicates significant effects of CASK silencing on p21 mRNA expression

After observing the effects of CASK silencing on increasing p21 and EGFR expressions, we are interested in understanding the role of EGFR in p21 expression. To this end, we treated cells with EGFR TKIs, gefitinib (10 µM), U0126 (10 µM), and AKTI (10 µM), in different culture conditions. First, cells were cultured in CM overnight before replacing with SF medium for the indicated time. We found that the protein expression level of p21 was significantly decreased by gefitinib, U0126, and AKTI, both in H1299 and PC9 shCASK cells (Fig. 9A). In another culture condition, cells were cultured in SF medium overnight before replacing with CM for the indicated time. Similarly, we found p21 expression was also significantly reduced by AKTI both in H1299 and PC9 shCASK cells (Fig. 9B). On the other hand, p21 mRNA expression level was reduced by gefitinib and U0126, but not by AKTI, both in H1299 and PC9 shCTL and shCASK cells (Fig. 9C). These findings indicate that EGFR-ERK and EGFR-AKT signaling mediate p21 expression via the transcription and post-transcription mechanisms, respectively. Furthermore, given that CASK exerts both kinase-dependent [9, 38, 39] and kinase-independent scaffolding functions [5, 40, 41], we used NR162, a CASK kinase inhibitor [42], to investigate its effect on p21 expression. Results showed that NR162 increased the expression levels of p21 and p-ERK, whereas the expression levels of EGFR and p53 were not affected in PC9 cells (Fig. 9D). These findings suggest that CASK’s regulation of EGFR and ERK-dependent p21 expressions relies distinctly on scaffold and catalytic activity, respectively.

### ERK inhibits EGF-induced EGFR degradation by delaying EGFR from entering late endosomes

**Fig. 10:**
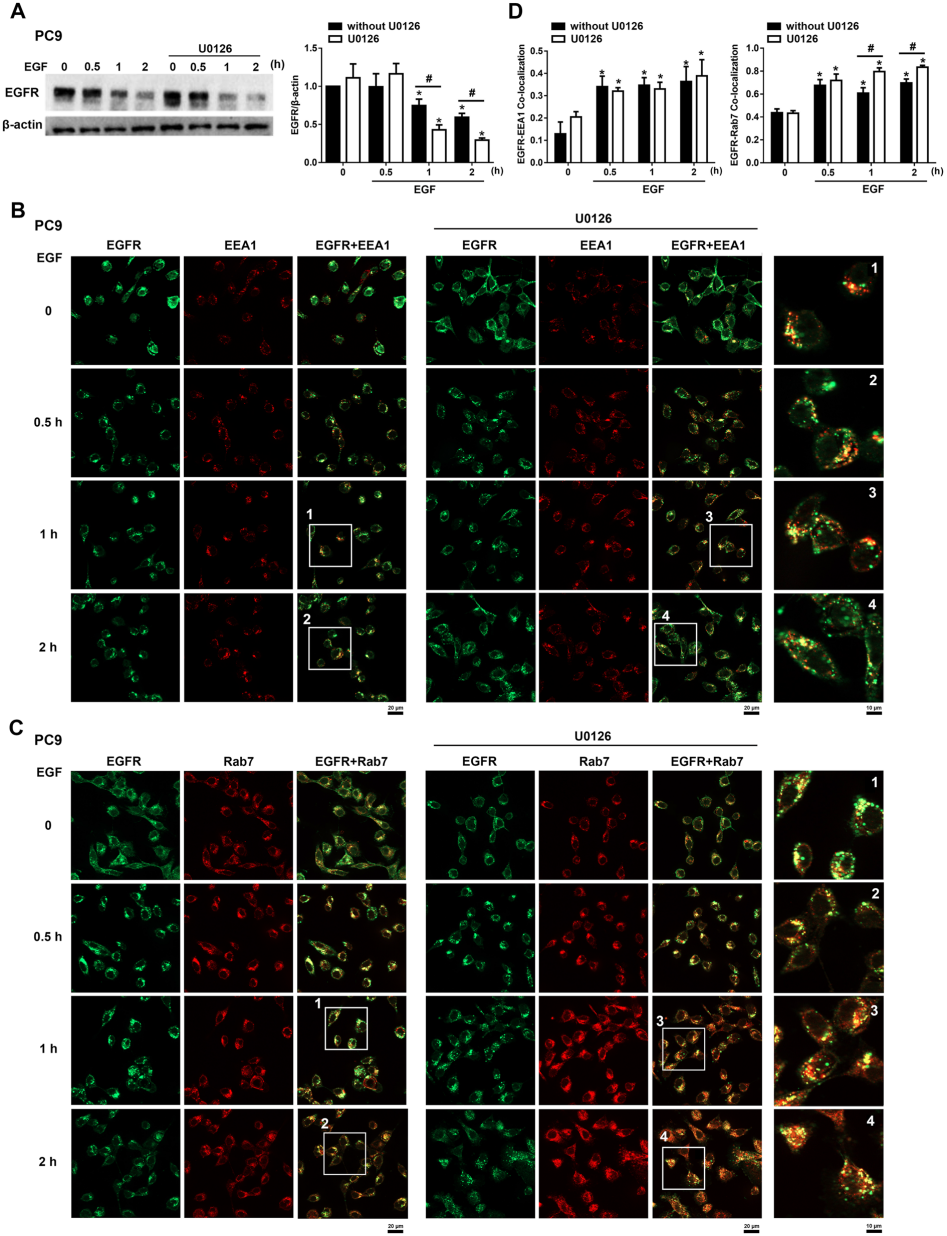
ERK inhibits EGFR trafficking to late endosomes. PC9 cells were cultured in SF medium overnight and then stimulated with EGF (50 ng/ml) in the absence or presence of U0126 (10 μM) for the indicated times. (**A**) EGFR protein levels were analyzed by immunoblotting. (**B, C**) The subcellular localization of EGFR with EEA1 (**B**) or Rab7 (**C**) was determined by immunofluorescence staining. Scale bars represent 20 μm. Enlarged views of boxed regions are shown on the right. (**D**) Statistical analysis of EGFR colocalization with EEA1 and Rab7 was presented as bar charts. Data are presented as the mean ± S.E.M. from at least three independent experiments. *p < 0.05 indicates significant effects of EGF on EGFR degradation (**A**) and its colocalization with EEA1 and Rab7 (**D**). #*p* < 0.05 indicates significant effects of U0126 on enhancing EGF-induced EGFR degradation (**A**) and on the colocalization of EGFR with Rab7 (**D**)

ERK activation was reported to regulate the EGFR endosomal trafficking pathway [43–45]. Since CASK silencing increased basal ERK activation and delayed EGFR degradation, we wondered whether ERK activity was involved in the action of CASK in regulating EGFR trafficking. Firstly, we found that EGF-induced EGFR degradation was enhanced by U0126 in PC9 shCTL cells (Fig. 10A). In addition, we conducted an immunofluorescence experiment to assess if U0126 affected the co-localization between EGFR and endosomal markers. We found that EGF treatment induced EGFR co-localization with EEA1, and this effect was not changed by U0126 treatment (Fig. 10B, D). Similarly, EGF treatment also induced EGFR co-localization with Rab7, while this effect was enhanced under U0126 treatment (Fig. 10C, D). These results suggest that ERK decreases EGF-induced EGFR degradation by delaying the trafficking of EGFR into late endosomes.

## Discussion

Lung cancer remains the leading cause of cancer-related mortality worldwide. Despite significant advances in the management of NSCLC, major limitations persist, and unresolved challenges continue to hinder progress [46]. Therefore, a deeper understanding of novel regulatory mechanisms driving disease progression is critical. CASK, a ubiquitously expressed scaffold protein with predominant functions in the brain, has only recently been implicated in cancer biology [6–10], and its roles in tumor progression remain poorly defined. In this study, we identified CASK as a novel regulator that promoted the growth of NSCLC cell lines (H1299 and PC9) by suppressing p21 expression. Silencing of CASK induced both p21 mRNA and protein levels, mediated by downstream EGFR signaling and a p53-independent pathway. Notably, CASK depletion impaired EGF-induced EGFR degradation by delaying ERK-dependent trafficking of EGFR to late endosomes. Furthermore, increased expression of the endogenous EGFR ligand TGF-α, driven by p53 and the autocrine loop of EGFR activity, contributed to the enhanced constitutive EGFR activation in CASK-silenced cells.

CASK is a multifunctional scaffold protein. Previous studies have demonstrated that the regulatory role of CASK in cell proliferation differs between normal and malignant contexts. CASK has been reported as a tumor promoter in liver [10], prostate [9], colorectal [6], pancreatic [8], and esophageal [47] cancers. In the present study, we extend these observations to NSCLC. We show that CASK is highly expressed in lung adenocarcinoma and that elevated expression correlates with poor OS and FP survival. Importantly, CASK upregulation is detectable at early stages of tumor development, underscoring its potential as a biomarker and prognostic factor in NSCLC. Complementary in vitro experiments in PC9 cells further suggest the essential role of CASK in cell proliferation. These findings reinforce the concept that CASK is a multifunctional molecule capable of regulating diverse cellular processes. This activity likely reflects its established role as a scaffold protein, supported by evidence of multiple interacting partners in the brain and its functions in synaptic plasticity, neurotransmitter release, and neurodevelopment [5, 40, 41].

Our study demonstrates that CASK negatively regulates p21 mRNA and protein expression in NSCLC. Although CASK serves as a tumor promoter in several tumor types, as mentioned above, the mechanisms underlying CASK-associated tumor progression remain incompletely defined. Current evidence is restricted to pancreatic and prostate cancer, where CASK promotes malignancy through activation of Notch and AKT signaling pathways, respectively [8, 9]. In contrast, reports on the regulation of normal cell growth by CASK are limited and suggest an opposing role. Rather than enhancing proliferation, CASK negatively regulates growth in non-malignant cells. For example, CASK knockdown in cultured keratinocytes and organotypic skin raft cultures accelerates cell cycling, accompanied by Myc upregulation and hyperphosphorylation of Rb [11]. Similarly, CASK suppresses endothelial ECV304 cell growth by enhancing E2A-dependent p21 transcription. Mechanistically, CASK interacts with Id1, an inhibitor of E2A, thereby increasing E2A-mediated activation of the p21 gene [12, 48]. These findings suggest that the mechanism of CASK action is highly cell type–dependent. This variability is further complicated by the dual role of p21 in cancer biology. In the nucleus, p21 functions as a tumor suppressor in NSCLC by inhibiting cyclin-CDK complexes, blocking Rb phosphorylation, repressing E2F target genes, and enforcing cell cycle arrest and senescence [49]. Conversely, when mislocalized to the cytoplasm via AKT-dependent phosphorylation, p21 inhibits apoptosis through interactions with caspases and other apoptotic regulators, thereby promoting survival and acting as a tumor promoter [50]. Thus, the differential regulation of p21 by CASK underscores its bidirectional functions in normal versus malignant cells and highlights the complex interplay between CASK and p21 in controlling cell growth and cancer progression.

The regulation of p21 protein expression is controlled at multiple levels, encompassing both transcriptional and post-translational mechanisms, to coordinate cell cycle arrest, DNA repair, and senescence [51, 52]. The canonical mechanism involves transcriptional activation by p53, which upregulates p21 gene expression in response to DNA damage and cellular stress, thereby inhibiting CDKs and halting cell cycle progression [53, 54]. Beyond p53, several transcription factors—including Sp1, E2A, STATs, and SMADs—modulate p21 gene expression under diverse signaling contexts. Among these, Sp1 plays a pivotal role as a direct promoter-binding factor, cooperating with SMADs, STATs, E2A, and c-Jun to drive p21 gene upregulation [54–57]. In this aspect, elevated p21 expression is often driven by upstream signals such as the EGFR–ERK pathway in different cell types. EGFR has been shown to contribute to a higher early p21 expression in keratinocytes [58, 59], fibroblasts [16], A431 cells [17], bronchial epithelial cells [60], squamous carcinoma cells [18], enterocytes [19], head and neck cancer [20], HCC [21], and glioblastoma cells [22]. Multiple transcription factors, including Sp1 [61–63], E2A [56], and c-Jun [64] that bind to the p21 promoter for serum-induced p21 expression have been documented. Similarly, the ERK-dependent increase of p21 mRNA expression was shown in NSCLC [65] and other cancer cell types [52, 66]. In this study, we also suggest that p21 expression is positively regulated by EGFR-ERK signaling in CASK-silencing NSCLC. Evidence for this notion is provided by the enhanced EGF-induced ERK phosphorylation observed upon CASK silencing, together with the marked suppression of p21 gene and protein expression by EGFR and ERK inhibitors. Another piece of evidence to support our observation on EGFR-dependent cell suppression is the effect of EGF in inducing apoptosis in EGFR-overexpressing cancer cells [67]. In this study, we also demonstrate that the CaMK activity of CASK contributes to suppression of the ERK–p21 axis in PC9 cells. Notably, there are currently no direct reports describing CaMK-mediated repression of p21 expression or inhibition of ERK signaling. Therefore, further investigation is warranted to address this issue in the future. In addition to the ERK pathway, AKT enhances p21 protein stability by phosphorylating Thr145 and Ser146 [68, 69]. In this study, we demonstrate that CASK silencing increases EGFR-mediated AKT activity, similar to its effect on ERK. Notably, inhibition of AKT suppresses p21 protein levels but does not affect p21 gene upregulation, suggesting that EGFR–AKT signaling contributes to p21 expression through post-transcriptional regulation.

p53 is a well-established transcriptional activator of p21, particularly under DNA damage conditions. Our findings suggest that CASK silencing-induced p21 gene expression is predominantly ascribed to a p53-independent mechanism. To clarify this notion, we conducted a reporter assay of p21 promoter activity. Although p53 binding site deletion markedly attenuates luciferase activity, CASK silencing can still increase the activity under such conditions. Likewise, p21 mRNA level is upregulated in shCASK cells upon treatment with PFT-α, a pharmacological inhibitor of p53. Mechanistically, PFTα inhibits p53 phosphorylation at Ser15 and Ser33 [70] and reduces the binding affinity of p53 to the p21 promoter, thereby increasing its transcriptional activity [71]. Moreover, in the p53-null H1299 cells [72], the p21 upregulation is similarly increased by CASK silencing. Taken together, these findings indicate that CASK silencing enhances p21 expression through EGFR/ERK-dependent but p53-independent transcriptional mechanisms, as well as EGFR/AKT-dependent post-transcriptional stabilization.

We speculate that transcription factor(s) beyond p53, particularly those binding to the − 1866 to − 180 region of the p21 promoter, may be regulated by CASK. In this context, previous studies have identified FOXA1/2 as key transcription factors capable of activating p21 transcription in p53-null H1299 cells through direct binding to two promoter regions located at − 596 to − 585 and − 451 to − 440 [73]. In addition, even though the outcome is different from the current study, CASK has been reported to upregulate p21 expression via the E2A transcription factor in human endothelial cells [12], suggesting that CASK may influence p21 transcription through multiple transcriptional regulators. Consistent with these observations, our p21 reporter assays demonstrated that luciferase activity remained elevated in CASK-silenced cells transfected with a p53-binding site-deleted construct that retains FOXA1/2 binding sites. This finding indicates that CASK-mediated regulation of p21 is, at least in part, independent of p53 and may involve alternative transcription factor networks. Taken together, these results raise the possibility that CASK modulates p21 expression through a FOXA1/2-related pathway or other non-p53-dependent mechanisms. Further studies will be required to delineate the precise regulatory interactions.

Although CASK silencing does not alter overall p53 protein abundance, the reduction of p21 expression by PFT-α, together with the diminished p21 promoter activity in the p53-binding site-deleted construct, indicates that the p53–p21 axis remains functionally engaged. Here, we found that CASK silencing increases p53 protein stability. MDM2 is a well-identified upstream ubiquitination signal for p53 degradation, and ERK contributes to MDM2 phosphorylation and activation [30]. Our current findings indicate that shCASK-mediated stabilization of p53 occurs independently of MDM2-driven proteasomal degradation. The major reason is the lack of a decrease in MDM2 expression in shCASK cells. Moreover, the efficacy of proteasomal degradation of p53, as indexed by MG132 and/or U0126 treatment, is similar in shCTL and shCASK cells. ERK was reported to upregulate MDM2 activity by phosphorylating MDM2 at Ser-166, thereby increasing the degradation of p53 [30]. These results argue against a major role for the canonical MDM2–proteasome axis in mediating the effect of CASK on p53 stability. We suggest that shCASK-mediated stabilization of p53 protein occurs independently of MDM2-driven proteasomal degradation of p53. Alternative mechanisms warrant further investigation in future studies.

In this study, we confirm that constitutive EGFR activation in NSCLC is sustained through a positive regulatory loop. Once EGFR is activated, downstream signals such as ERK and AKT feedback to reinforce EGFR activity by upregulation of EGFR itself and/or the expression of EGFR ligands, as well as preventing EGFR downregulation. This sustained EGFR activation represents a hallmark of NSCLC progression and a major mechanism underlying EGFR-TKI resistance [74, 75]. Notably, this process is negatively regulated by CASK, which suppresses endogenous gene expression of the key EGFR ligand TGF-α and EGFR, while simultaneously promoting EGFR degradation. Pharmacological inhibitor studies further reveal that EGFR downstream signal ERK contributes to the upregulation of EGFR gene expression, whereas EGFR, ERK, AKT, and p53 are involved in driving TGF-α expression. The latter observation is consistent with previous reports showing that ERK and AKT signaling enhance TGF-α expression in NSCLC, establishing an autocrine positive feedback loop that sustains EGFR activation and promotes tumor progression [76, 77]. In addition, p53 has been reported to indirectly regulate TGF-α expression and activity by modulating EGFR pathway [78].

CASK is a multidomain scaffold protein that plays a critical role in trafficking and plasma membrane localization of diverse proteins, including EGFR [14], DLG1, LIN7C [79], AMPAR, NMDAR [80], and Kir2 channels [81]. In *C. elegans*, the LIN-2 (CASK)/LIN-7 (Lin7A-C)/LIN-10 complex, localized at the Golgi, is required for LET23 (EGFR) targeting to the basolateral membrane of vulval precursor cells [14, 82]. Dysregulated ERK–EGFR trafficking has been reported in NSCLC, where it sustains signaling, promotes tumor progression, and contributes to resistance against EGFR-targeted therapies. In our study, we found that CASK silencing not only increases EGFR gene and protein expression but also enhances EGFR stability in NSCLC cells. Mechanistically, CASK regulates EGFR trafficking at the step of transport to the late endosome, without affecting receptor internalization from the plasma membrane to the early endosome. Notably, shCASK-induced ERK activation delays EGFR entry into late endosomes, thereby inhibiting EGF-induced EGFR degradation and favoring receptor recycling back to the plasma membrane. Consistent with previous reports, EGF-activated EGFR and ERK localize to endosomal compartments, where signaling modulates trafficking by phosphorylating regulators and influencing endosomal sorting, recycling, and degradation [43–45, 83]. ERK-dependent phosphorylation of EGFR at T669 has been shown to inhibit ligand-induced degradation [83], while Xiao and Schmid demonstrated that ERK activity prevents efficient progression of EGFR to late endosomes and lysosomes [84]. Our findings extend these observations by identifying CASK as a negative regulator of ERK-dependent inhibition of EGFR trafficking to the late endosome. This highlights CASK as a novel modulator of endosomal vesicle trafficking, with its regulatory effects likely dependent on cellular context. Moreover, our findings using a pharmacological kinase inhibitor of CASK further suggest a CaMK-independent action of CASK in regulating EGFR expression and activation. Therefore, future studies should aim to identify novel scaffold molecules of CASK beyond EGFR and to elucidate in detail the mechanisms underlying their roles in cellular responses.

Our data in this study suggest that CASK plays an important role in cytoskeletal regulation. RNA-Seq analysis revealed that CASK is involved in controlling actin cytoskeleton–associated cell morphology and functions, including focal adhesion, tight junctions, adherens junctions, and migration. This observation in NSCLC is consistent with previous reports showing that CASK regulates actin organization, microtubule dynamics, adhesion, and cell migration in other cell types [85, 86]. When examining the role of CASK in mitosis, we found that CASK silencing enhances EpoB–induced α-tubulin acetylation in NSCLC cells. The absence of effects of PFTα and UC2288 indicates that this regulation is independent of EGFR signaling, p21, and p53 activity in microtubule dynamics, suggesting that CASK can regulate cell cycle beyond p21. This finding also creates a new direct action of CASK in microtubule dynamics, which is essential for cell mitosis. The crucial role of CASK in regulating microtubule dynamics has been highlighted by its scaffolding function as a component of the microtubule-associated trafficking complex, where it participates in NMDAR trafficking [87] and sorting [88]. Although CASK has been shown to interact with Dlg1 and regulate microtubule organization and spindle orientation in mammalian epithelia [89], there is no strong evidence that Dlg1 directly controls α-tubulin acetylation. To date, no direct reports have demonstrated how CASK regulates α-tubulin acetylation. Future studies will be required to elucidate the molecular mechanisms by which CASK influences acetyl-modification of α-tubulin.

In this study, we observed cell-type-specific effects related to CASK silencing in PC9 and H1299 cells. These include gene regulation and CDK1. H1299 cells harbor wild-type EGFR and are deficient in p53. In contrast, PC9 cells carry activating EGFR mutations and demonstrate greater drug sensitivity [90]. We therefore speculate that the different effects of CASK silencing on the expression of COL6A3, PLA2G2F, PIK3R3, FN1, and CDK1 in H1299 cells compared to PC9 cells may be attributable to the underlying genetic difference in EGFR. Among the top genes affected by CASK, GNG7 is a tumor suppressor in lung adenocarcinoma and suppresses lung cancer progression by inhibiting E2F1 [91]. Increased MYLK expression is observed in stage III and IV in NSCLC and contributes to metastasis [92]. Moreover, PIK3R3 [93], COL6A3 [94, 95], and fibronectin [96] promote cancer cell proliferation and metastasis. The relationship between CASK and these molecules in NSCLC tumorigenesis needs further investigation.

Current knowledge of disease-related CASK gene mutations is largely confined to neuronal disorders. For example, a splice mutation (c.2521-2A>T) in the GK domain [2], a nonsense mutation (p.R639X) in the SH3 domain [97], and a missense mutation (p.R28L) in the CaMK domain [98] have been linked to X-linked intellectual disability syndromes, including microcephaly, optic atrophy, and FG-like syndrome. In contrast, the association between CASK gene mutations and cancer progression remains poorly defined. In this study, we identified G-to-T substitutions and missense mutations as the most frequent *CASK* alterations in lung cancers, with the majority of mutation sites localized to the CaMK domain. However, given the limited sample size available from the cBioPortal database, no definitive conclusions can yet be drawn regarding the impact of CASK mutations on OS in NSCLC. This question remains an intriguing avenue for future investigation.

## Conclusions

We demonstrate for the first time the crucial role of CASK in NSCLC growth and delineate its underlying mechanisms in promoting cancer cell proliferation. CASK suppresses p21 expression by attenuating EGFR/ERK-dependent transcriptional regulation in a p53-independent manner, as well as EGFR/AKT-dependent post-transcriptional stabilization. Moreover, the positive autocrine loop of EGFR activation in NSCLC, driven by ERK- and/or AKT-dependent induction of TGF-α and EGFR expression, is fine-tuned by CASK. Specifically, CASK negatively regulates EGFR activity by repressing TGF-α and EGFR expression and by mitigating the ERK-dependent delay in EGFR trafficking to late endosomes for degradation. Collectively, these findings highlight CASK as a potential biomarker and prognostic indicator in NSCLC.

## Supplementary Information

Supplementary Material 1

## Data Availability

All data generated or analysed during this study are included in this published article.

## Abbreviations

CASK: Calcium/calmodulin-dependent serine protein kinase
EGFR: Epidermal growth factor receptor
NSCLC: Non–small cell lung cancer
MAGUK: Membrane-associated guanylate kinase
CaMK: Ca^2+^/calmodulin-dependent protein kinase
GUK: Guanylate kinase
CDK: Cyclin-dependent kinase
SH3: Src homolog 3
EPS-8: EGFR substrate protein 8
HCC: Hepatocellular carcinoma
BafA1: Bafilomycin A1
BSA: Bovine serum albumin
PBS: Phosphate-buffered saline
FBS: Fetal bovine serum
PFT-α: Pifithrin-α
CHX: Cycloheximide
EpoB: Epothilone B
DEGs: Differentially expressed genes
TCGA: The cancer genome atlas
KEGG: Kyoto encyclopedia of genes and genomes
GEO: Gene expression omnibus
GO: Gene ontology
COSMIC: Catalogue of somatic mutations in cancer
PI: Propidium iodide
RT-PCR: Real-time polymerase chain reaction
BrdU: Bromodeoxyuridine
OS: Overall survival
FP: First progression
CM: Complete medium
SF: Serum-free
TGF-α: Transforming growth factor-α
AREG: Amphiregulin
EREG: Epiregulin
