# Supplementary material for: CASK promotes non-small cell lung cancer growth through coordinated regulation of EGFR expression, trafficking, and p21 expression
Source: J Biomed Sci. 2026 May 9;33:49. doi: 10.1186/s12929-026-01252-z (PMC13156893; doi:10.1186/s12929-026-01252-z)
Supplement: Supplementary file 1 — Supplementary Material 1 [file 12929_2026_1252_MOESM1_ESM.docx]

**Supplementary Table 1** List of primer sequences used in this study

| Target mRNA | Forward primer (5’→3’) | Reverse primer (5’→3’) |
| --- | --- | --- |
| p21 | TGGAGACTCTCAGGGTCGAAA | GGCGTTTGGAGTGGTAGAAATC |
| TP53 | GCCCACTTCACCGTACTAACCA | GAAACTACCAACCCACCGACCA |
| EGFR | GACCTCCATGCCTTTGAGAA | GCTGACGACTGCAAGAGAAA |
| TGF**-**α | GGTCCGAAAACACTGTGAGTGG | CAAACTCCTCCTCTGGGCTCTT |
| EGF | TGCGATGCCAAGCAGTCTGTGA | GCATAGCCCAATCTGAGAACCAC |
| AREG | GCACCTGGAAGCAGTAACATGC | GGCAGCTATGGCTGCTAATGCA |
| EREG | CTTATCACAGTCGTCGGTTCCAC | GCCATTCAGACTTGCGGCAACT |
| GNG7 | CTTGGTTTCGGGATCTCGGT | CCCCGTTGTTCAGAGAGCTT |
| MYLK | TGAGTCCAACCCTCGTGTCTA | AAAGCTTGGGACACATCTTCTTCA |
| COL6A3 | AACATCGGCACTTGCCCTTA | AGAGTCTTGTGCTGCTTGCT |
| PLA2G2F | CTCCAGGCAACACAGAGGAG | GGTGGCTTCAGTGGTTTTCG |
| PIK3R3 | CTGGAGGGAGGTGATGATGC | GCATCTCGGACCAAGAAGGT |
| FN1 | TTCTGGTCAGCAACCCAGTG | GCCTCTGCTGGTCTTTCAGT |
